# Exercise‐specific plasma proteomic signatures in racehorses: Candidates for training adaptation and peak load monitoring

**DOI:** 10.1002/evj.70146

**Published:** 2025-12-29

**Authors:** Jowita Grzędzicka, Bianka Świderska, Ewa Sitkiewicz, Izabela Dąbrowska, Olga Witkowska‐Piłaszewicz

**Affiliations:** ^1^ Department of Large Animal Diseases and Clinic, Institute of Veterinary Medicine Warsaw University of Life Sciences Warsaw Poland; ^2^ Mass Spectrometry Laboratory, Institute of Biochemistry and Biophysics Polish Academy of Sciences Warsaw Poland

**Keywords:** equine sports medicine, exercise adaptation, horse, inflammation, LC–MS, metabolic stress, oxidative stress, proteomics, TMTpro labelling, training biomarkers

## Abstract

**Background:**

Racehorses undergo profound physiological changes with training and competition, but current biomarkers inadequately capture the complex molecular dynamics of exercise. This study aimed to identify novel plasma biomarkers of training adaptation and peak load using high‐throughput proteomics.

**Objectives:**

We hypothesised that systematic training and racing induce distinct plasma proteomic signatures, enabling the discovery of candidate biomarkers linked to training status, oxidative stress, inflammation and metabolic remodelling.

**Study Design:**

In vivo longitudinal study.

**Methods:**

Forty‐nine Arabian and Thoroughbred racehorses underwent standardised high‐intensity training. Plasma samples were collected at rest, immediately post‐exercise and after recovery during three phases: initial training (T1), mid‐season conditioning (T2) and race‐phase (R). In total, 314 samples were analysed using tandem mass tags based quantitative proteomics and Orbitrap mass spectrometry. Protein abundance changes were assessed with multiple‐testing correction (*q* < 0.05), and pathway enrichment was performed using STRING and ShinyGO.

**Results:**

Proteomic responses differed by phase. T1 showed broad activation of inflammatory (S100A8/A9), antioxidant (superoxide dismutase 1, catalase) and metabolic proteins (glucose‐6‐phosphate dehydrogenase, phosphoglycerate kinase 1). T2 displayed a more refined profile with remodelling and redox regulators (decorin, thymosin β4, glutathione S‐transferase). Racing elicited the strongest response, with over 100 up‐regulated proteins linked to energy metabolism, oxidative defense and cytoskeletal adaptation. Several proteins: including S100A8, thymosin β4, prothymosin‐α, cofilin‐1 and lipocalins, were consistently modulated across phases, highlighting their biomarker potential.

**Main Limitations:**

Breed imbalance and incomplete follow‐up sampling may affect generalisability. Validation in larger, diverse cohorts with targeted assays is required.

**Conclusions:**

This study identifies a panel of promising plasma proteins as candidate biomarkers of exercise adaptation and overload in racehorses. These findings may support improved monitoring of performance, training load and early detection of overtraining in equine athletes.

## 1 INTRODUCTION

Racehorses and sport horses undergo significant physiological adaptations to exercise, driven by both evolutionary processes and selective breeding. Regular training enhances muscle mass, improves aerobic capacity and promotes a shift towards muscle fibres that are more resistant to fatigue,^1^ enhanced glycogen storage,^2^ and improved cardiovascular efficiencies.^3^ However, the molecular mechanisms behind these adaptations remain poorly understood. Traditional methods of assessing exercise adaptation, such as heart rate, lactate levels and standardised exercise tests, provide useful data but fail to capture the complex metabolic and stress‐related changes in the body. Therefore, there is a need to identify new biomarkers that can more accurately assess training status and detect subclinical signs of overload.^4^, ^5^

Proteomics, the large‐scale study of proteins, is a powerful approach for examining the molecular changes induced by exercise. Proteins lie at the core of biological function, and profiling the plasma proteome enables a nuanced view of the functional adaptations elicited by training.^6^ Modern proteomic techniques, particularly mass spectrometry, allow for the detection of hundreds of proteins simultaneously, providing a deeper understanding of metabolic, inflammatory and immune responses that might be missed by traditional assays. Previous proteomic studies have demonstrated that exercise alters the expression of proteins related to energy storage, inflammation and oxidative metabolism in racehorses.^7^, ^8^, ^9^, ^10^ Moreover, proteomics holds promise for identifying biomarkers of overtraining,^11^, ^12^ such as an increase in alpha‐1‐antitrypsin, which could indicate training overload.^8^

Despite the advances in proteomics, there is a lack of research focusing on the changes in protein production in horses monitored during various phases of racing training. This gap underscores the need for a comprehensive study that tracks these changes across the different training phases. Our study aims to address this gap by using proteomics to investigate the molecular basis of equine exercise adaptation. By identifying proteins that reflect the horse's response to physical stress during training and heavy exercise, we hope to better understand the molecular processes behind exercise adaptation and, importantly, help identify biomarkers for performance and training peak load.

## 2 MATERIALS AND METHODS

### 2.1 Animals

The study involved 49 racehorses, consisting of 37 Arabians and 12 Thoroughbreds (15 mares, 33 stallions and 1 gelding), aged 2–7 years (median age: 3 years) (Table S1). All horses were trained by the same experienced trainer, following a consistent high‐intensity training regimen. Each horse was housed in individual stables with proper bedding and had the opportunity for outdoor turnout, weather permitting. They received a standardised diet that included high‐quality hay, commercial concentrate feed and vitamin‐mineral supplements, with unrestricted access to fresh water. All horses were clinically healthy, dewormed and vaccinated prior to the racing season and remained under continuous veterinary supervision throughout the study.

The sample size was determined based on previous proteomic studies in horses and other large animals, which revealed substantial inter‐individual variability and complex patterns of protein regulation. These factors necessitated the use of relatively large cohorts, as in earlier research: 16 horses,^11^ 13 horses,^10^ and 12 horses in comparable studies.^12^ While earlier studies often included 12–16 subjects per group, recent evidence suggests enhanced statistical power with at least 12–20 paired observations in within‐subject designs.^10^, ^11^, ^12^, ^13^ Assuming a large effect size (Cohen's *d* ≈ 1.0) for key proteins related to immune and stress responses (e.g., afamin, apolipoprotein E and protein alpha‐1‐microglobulin/bikunin precursor),^8^, ^10^, ^11^ power analysis using G*Power (v3.1) for a two‐tailed, paired *t*‐test indicated that 16 horses (i.e., 16 paired pre‐ and post‐training samples) would be sufficient to detect significant differences with 80% power at *α* = 0.05.

The training regimen was conducted six days a week, weather permitting. Each session began with a 10‐min warm‐up walk under saddle, followed by 800 m of trotting and another 800 m of relaxed cantering. The conditioning phase included 1400–2400 m of moderate cantering (16–22 s per 200 m), followed by a high‐intensity gallop over 500–1200 m at 80–100% of maximum speed (13–15 s per 200 m). Each session concluded with a 30–40 min cool‐down using a mechanical walker. High‐intensity galloping was scheduled every 5–10 days to enhance performance.

### 2.2 Blood sampling

Blood samples were collected as part of routine veterinary monitoring to assess baseline and exercise‐induced changes in haematological and biochemical parameters related to health and performance. Samples were taken at three time points: at rest (p0), immediately post‐exercise (p1) and after a 30–40 min recovery (p2). Blood was collected during three phases of the training season: start‐of‐season training for the current campaign called also initial training (T1; *n* = 49), conditioning (T2; *n* = 44) and the race‐phase (R; *n* = 37), which included official races or high‐intensity gallops. Most horses participated in all three stages, with some excluded due to early cessation, sale or sample quality issues. This was a longitudinal, within‐subject study with repeated sampling across training phases; no allocation of animals to intervention arms was performed.

At T1, 128 plasma samples were analysed (p0 *n* = 49, p1 *n* = 49, p2 *n* = 30); at T2, 115 samples (p0 *n* = 44, p1 *n* = 43, p2 *n* = 25); and during R, 74 samples (p0 *n* = 37, p2 *n* = 35). Venous blood was aseptically drawn from the jugular vein using sterile, single‐use needles. To minimise artefactual changes due to cellular release, venipuncture was performed with minimal stasis, and samples were immediately inverted. Haematology samples were collected in EDTA tubes, and biochemical samples in serum tubes (Vacutainer, BD). Plasma for proteomic analysis was separated within 4 h and visually inspected for haemolysis; samples with haemolysis were excluded. Plasma was stored under controlled conditions until processing.

### 2.3 Sample preparation

A total of 314 horse plasma samples were subjected to proteomics analysis. An internal standard (IS) sample was prepared by pooling equal volumes of plasma from each horse.

Albumin was depleted from both individual plasma samples and the IS using a modified reducing agent‐mediated precipitation method. Briefly, 5 μL of plasma was mixed with 20 μL of phosphate‐buffered saline and 15 μL of 500 mM tris(2‐carboxyethyl)phosphine to reach a final concentration of 187.5 mM. Samples were vortexed at 1000 rpm for 1.5 h at 25°C, followed by centrifugation at 4000 g for 1.5 h at 25°C. Next, 20 μL of the diluted, albumin‐depleted plasma was transferred to a new deep‐well 96‐well plate for digestion using a modified SP3 protocol.^14^ Magnetic beads were prepared by combining equal volumes of Sera‐Mag Carboxyl hydrophilic and hydrophobic particles (Cytiva). After washing, beads were resuspended to a final concentration of 10 μg/μL. To each sample, *n*‐dodecyl‐d‐maltoside detergent to a final concentration of 1%, formic acid to a concentration of 0.01%, and 50 μL bead suspension were added, followed by binding with 90% acetonitrile (ACN), washing with 80% ethanol and methyl methanethiosulphonate (30 mM in water) treatment to block cysteines. After additional washing with 80% ethanol and 100% ACN, proteins were digested overnight at 37°C in 100 mM triethylammonium bicarbonate buffer with 1:50 enzyme:protein ratio trypsin (Promega). Peptide concentrations were measured using the Pierce Quantitative Colorimetric Peptide Assay (Thermo Scientific) according to the manufacturer's instructions.

### 2.4 Randomisation and batch allocation

Potential batch and run‐order bias were minimised by randomising plasma samples at two levels using a computer‐generated sequence (Excel RAND): (i) allocation to 96‐well plate positions during depletion/digestion and (ii) allocation to TMTpro sets and reporter channels, with stratification by session (T1/T2/R) and timepoint (p0/p1/p2) to maintain a balanced distribution across sets. Each set included a common IS (reporter 126) to support cross‐set normalisation; remaining batch effects were evaluated by principal component analysis (PCA) and adjusted with ComBat as described. We also evaluated alternative normalisation and correction strategies, including Limma‐based adjustment and Internal Reference Scaling correction based on the IS channel signals. The results obtained with each method were systematically compared using PCA to inspect sample clustering, multi‐scatter plots with Pearson correlation to assess reproducibility, boxplots to examine median signal alignment and distribution spread, and within‐sample histograms to detect potential distortions or artefacts in the data. Controlled sample randomisation ensured that both time point and session biological variations were retained following ComBat batch‐adjusted normalisation.

### 2.5 TMT labelling and fractionation

Peptides from each sample were labelled with TMTpro 18plex reagents (Thermo Fisher Scientific) according to the manufacturer's protocol. In total, 19 independent TMTpro sets were prepared, covering 314 individual samples and 19 IS samples. The 126‐reporter channel was reserved for the IS in each set. For labelling, 22 μg of peptide digest from each sample and IS was reacted with 0.4 mg of TMTpro reagent dissolved in anhydrous ACN. The reaction was quenched with hydroxylamine. Labelled peptides within each set were combined, desalted using reversed‐phase solid‐phase extraction cartridges (Waters), dried in a SpeedVac concentrator and fractionated by high‐pH reversed‐phase chromatography on Acquity ultra‐performance liquid chromatography H‐class system (Waters). Separation was carried out on an XBridge Peptide BEH C18 column (4.6 × 250 mm, 130 Å, 5 μm, Waters) at a flow rate of 0.8 mL/min. Mobile phases consisted of water (A), ACN (B) and 100 mM ammonium hydroxide (C). The percentage of phase C was kept constant at 10% throughout the gradient. Fractions were collected every 1 min starting from the third minute, and peptide elution was monitored at 214 nm using a UV detector. Ninety‐six fractions were collected for each set and subsequently concatenated by combining the first 32 and the last 32 fractions, resulting in 64 final fractions. Samples were dried in a SpeedVac concentrator and reconstituted in 0.1% formic acid prior to liquid chromatography‐tandem mass spectrometry (LC–MS/MS) analysis.

### 2.6 Mass spectrometry

Fractions were analysed using an Evosep One system (Evosep Biosystems) directly coupled to an Orbitrap Exploris 480 mass spectrometer (Thermo Fisher Scientific). For each fraction, 2 μg of peptides were loaded onto disposable Evotips C18 trap columns (Evosep Biosystems) according to the manufacturer's protocol. After washing, peptides were eluted and separated on an EV1106 analytical column (Dr Maisch C18 AQ, 1.5 μm beads, 150 μm ID, 15 cm length, Evosep Biosystems) using the 88 min gradient method (15 samples/day) at a flow rate of 220 nL/min. Data acquisition was performed in positive ion mode using a data‐dependent acquisition method optimised for TMTpro analysis. Full mass spectrometry (MS) scans (MS1) were acquired at 60000 resolution with a normalised automatic gain control (AGC) target of 300%, an automatic maximum injection time and a scan range of 300–1700 m/z. MS/MS scans (MS2) were acquired at 30000 resolution in TurboTMT mode with a Standard AGC target, an automatic maximum injection time, selection of the top 25 precursors within a 1.2 m/z isolation window and a Precursor Fit threshold of 70%. Dynamic exclusion was set to 20 s with a mass tolerance of ±10 ppm. Precursor ions above an intensity threshold of 5 × 10^3^ were selected and fragmented by higher‐energy collisional dissociation with normalised collision energy of 30%. The spray voltage was set to 2.1 kV, with a funnel RF level of 40 and a capillary temperature of 275°C.

### 2.7 Data analysis

Raw data were processed with MaxQuant (v2.5.0.0) using the *Equus caballus* Uniprot reference proteome (1 entry per gene, 21,432 entries, version 04.2025) and the MaxQuant contaminants database.^15^ Search parameters were as follows: enzyme trypsin, maximum 2 missed cleavages, minimum peptide length 7 aa, first‐search tolerance 20 ppm, main‐search 4.5 ppm, reporter‐ion tolerance 0.003 Da, fixed methylthio (C) and variable oxidation (M), a false discovery rate‐adjusted (FDR) 1% at peptide‐spectrum match and protein levels, ‘isobaric match between runs’ on.

Protein groups and quantitative data were further processed in Perseus (v1.6.15).^16^ Reverse hits, contaminants and site‐only identifications were excluded. Data were normalised using the median‐based loading factor method, log2‐transformed and filtered (proteins present in ≥50% of samples retained). Remaining missing values were imputed from a down‐shifted normal distribution (width 0.3, down‐shift 1.8) for each sample separately. Batch effects across tandem mass tags (TMT) sets were corrected using the ComBat algorithm (R package), with performance evaluated via distribution plots, correlations and PCA (Figure S1), as described in Section 2.4.

Statistical analyses used paired two‐sample *t*‐tests (horse‐paired design) across the full dataset. Differentially expressed proteins were defined as those meeting *q* ≤ 0.05 and fold change |FC| ≥ 1.2 (log2 FC 0.26). Differential abundance was defined primarily by FDR *q*‐value ≤0.05, while FCs were reported to facilitate biological interpretation and were not used as an additional criterion for statistical significance.

**FIGURE 1:**
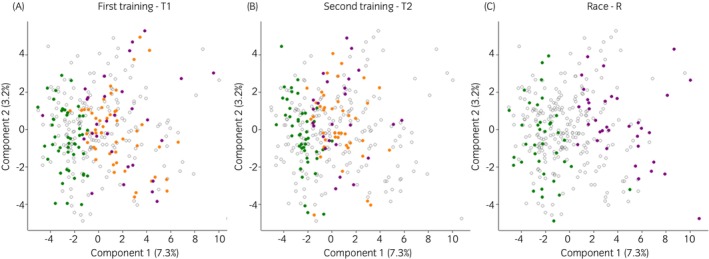
Principal component analysis (PCA) summarising the major sources of variation in the dataset, shown as scatter plots of the first two components (PC1 vs. PC2). (A) T1—initial training session, (B) T2—mid‐season conditioning session and (C) R—race‐phase. Colours indicate sampling timepoints relative to exercise: green p0P0 (pre‐training), orange p1 (immediately post‐training) and purple p2 (30 min post‐training). Each dot represents an individual sample. Clustering reflects similarity of overall profiles, and separation along PC1/PC2 indicates timepoint‐dependent differences.

Unless stated otherwise, FC is reported as a signed ratio with the convention ‘positive FC = higher abundance in the first‐named condition of the contrast; negative FC = higher abundance in the second’. Formally, for contrast A versus B: FC (A, B) = median (A/B) if A ≥ B, otherwise—median (B/A). As an effect‐size threshold, we use |FC| ≥ 1.20. Statistical filtering proceeds in two steps: first FDR *q* ≤ 0.05 within each contrast, then restriction to |FC| ≥ 1.20; we report counts after each step where relevant. Reported *q*‐values are adjusted within contrasts; because multiple contrasts are analysed, results across contrasts should be interpreted descriptively with this multiplicity in mind. A global overview of variance structure is shown by PCA (Figure 1) and a scree plot for this (Figure S2).

Qualitative conclusions were not contingent upon applying any specific FC threshold; instead, effect sizes are illustrated in the corresponding figures (volcano plots), which demonstrate that the sentinel proteins display robust, non‐marginal changes in the relevant phases.

We control the false discovery rate (Benjamini–Hochberg) within each contrast and define significance as *q* ≤ 0.05 together with |FC| ≥ 1.20. All inferential claims are based on *q*‐values (Tables S2 and S3).

### 2.8 Functional enrichment and network analysis

Functional enrichment analyses were performed using ShinyGO v0.82 (http://bioinformatics.sdstate.edu/go/) and STRING v12.0 (https://string-db.org). Gene ontology (GO) (Biological Processes, Molecular Functions, Cellular Components) and Kyoto Encyclopedia of Genes and Genomes (KEGG) pathway enrichment were assessed separately for up‐ and down‐regulated protein sets.

Enrichment was conducted using the default *Homo sapiens* background for annotation depth. Results were replicated with Equus ↔ Human ortholog mapping in g:Profiler to confirm robustness. Multiple‐testing correction was applied, and GO/KEGG terms were considered significant at *q* ≤ 0.10 (10% FDR).

STRING was further used to construct protein–protein interaction (PPI) networks, focusing on high‐confidence interactions (>0.7). Hub proteins were defined as the top 10% of nodes ranked by degree centrality; additional centrality measures (betweenness, closeness) were also calculated and reported to ensure robustness. Degree ranks are indicated in the corresponding STRING figures to facilitate interpretation. Functional modules were identified under default clustering, and subcellular localisation was inferred to assess compartment‐specific regulation. In total, we analysed 314 individual plasma samples organised into 19 TMTpro‐18plex sets. Each set was fractionated into 64 LC–MS runs, which yielded ~1216 raw files in addition to QC runs.

### 2.9 Candidate ranking

Candidate biomarkers were selected and ranked according to predefined criteria: (i) statistical robustness (paired design, FDR *q* ≤ 0.05), (ii) effect size (|FC| ≥ 1.20, used for interpretation rather than hypothesis testing), (iii) consistency across timepoints (directionally stable in ≥2 contrasts), (iv) network centrality (top 10% by degree within STRING high‐confidence networks, score >0.7; betweenness and closeness assessed for robustness), (v) technical robustness (≥2 unique peptides and detection in ≥50% of samples after filtering), (vi) biological plausibility in exercise physiology (literature evidence) and (vii) artefact‐risk auditing (haemolysis/red blood cell (RBC) admixture exclusion). To account for TMT ratio compression, emphasis was placed on *q*‐values and directional consistency rather than large FCs alone, with the expectation that targeted assays may yield higher effect sizes. The selected candidates are summarised in Table 1.

**TABLE 1:** Candidate panel of plasma proteins for monitoring training adaptation and peak physiological strain.

Protein	Direction (T1/T2/R; p2)	Typical FC range^a^	Function	Artefact risk	Validation priority
S100A8	↑ T1 p1/p2; ↑T2; ↑ R p2	1.6–2.0	Alarmin; neutrophil activation	Low–moderate (secreted/EV)	High
S100A9	↑ T1 p1/p2; ↑T2; ↑ R p2	1.5–1.9	Alarmin; innate immunity	Low–moderate (secreted/EV)	High
TMSB4	↑ T1/T2; ↑ R p2	1.3–1.9	Actin dynamics; repair	Low	High
PTMA	↑ T1; ↑ R p2	1.7–2.3	Cell growth; chromatin	Low	Medium
PGK1	↑ T1/T2; ↑ R p2	1.4–1.6	Glycolysis	Moderate (RBC‐susceptible)	Medium
G6PD	↑ T1/T2; ↑ R p2	1.3–1.6	PPP; NADPH/redox	Moderate (RBC‐susceptible)	High
SOD1	↑ T1; ↑ R p2	1.2–1.5	Antioxidant defense	Moderate (RBC‐susceptible)	Medium
Catalase	↑ T1; ↑ R p2	1.2–1.5	Antioxidant defense	Moderate (RBC‐susceptible)	Medium
CFL1	↑ T1/T2; ↑ R p2	1.3–1.7	Actin cytoskeleton	Low	Medium
Decorin (DCN)	↑ T1/T2; ↑ R p2	1.2–1.6	ECM/modulation	Low	Medium

Abbreviations: CFL1, cofilin‐1; ECM, extracellular matrix; G6PD, glucose‐6‐phosphate dehydrogenase; NADPH, nicotinamide adenine dinucleotide phosphate; PGK1, phosphoglycerate kinase 1; PTMA, prothymosin‐α; RBC, red blood cell; SOD1, superoxide dismutase 1; TMSB4, thymosin β4.

^a^ Fold change ranges are indicative and used for interpretation/ranking only (|ρ| < 0.20 Low; 0.20–0.40 Moderate; ≥0.40 High).

As this study was designed as a discovery‐phase proteomic screen, candidate selection followed established biomarker‐development frameworks in which untargeted LC–MS/MS data are sufficient for the discovery stage, while targeted verification parallel reaction monitoring (PRM) or enzyme‐linked immunosorbent assay (ELISA) is reserved for subsequent phases once the most promising markers have been nominated. This approach is consistent with current best‐practice guidance and avoids premature validation using non‐optimised immunoassays, which may introduce bias rather than increase confidence.^17^, ^18^

## 3 RESULTS

### 3.1 Identification of proteins with significant changes following initial training (T1)

**FIGURE 2:**
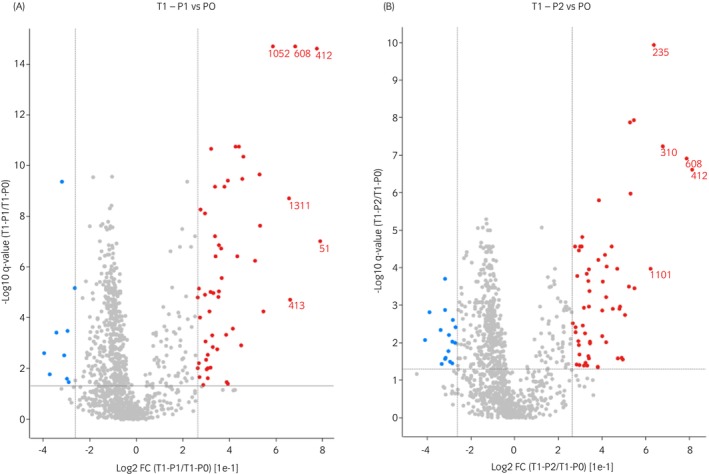
Volcano plot showing differentially abundant plasma proteins following the initial training session (T1). Protein numbers on the plot indicate IDs of proteins with |FC| > 1.5, corresponding to the identifiers listed in Table S2. (A) Significantly up‐regulated proteins (post‐exercise vs. pre‐exercise) (red) include PPIA (ID 1052), S100A8 (ID 412), S100A9 (ID 608), PGK1 (ID 413), PTMA (ID 51) and DNC (ID 1311), reflecting early activation of redox metabolism and cytoskeletal remodelling. (B) Strongly up‐regulated proteins (post‐recovery vs. pre‐exercise) include S100A8 (ID 412), S100A9 (ID 608), S100A12 (ID 1101), NUCB1 (ID 235) and LCN9 (ID 310), representing innate immune activation and lipid‐binding responses. Blue dots represent down‐regulated proteins. DNC, dynactin subunit 1; NUCB1, nucleobindin‐1; PGK1, phosphoglycerate kinase 1; PPIA, peptidyl‐prolyl *cis*–*trans* isomerase; PTMA, prothymosin‐α.

Relative to baseline (p0), p1 versus p0 yielded 529 proteins with *q* ≤ 0.05, of which 63 passed |FC| ≥ 1.2 (54 up, 9 down). In p2 versus p0, 475 proteins met *q* ≤ 0.05; 75 passed |FC| ≥ 1.2 (59 up, 16 down). In the direct p1 versus p2 comparison, 14 proteins met *q* ≤ 0.05 and |FC| ≥ 1.2 (8 higher at p1, 6 higher at p2). Session‐level volcano plots are shown in Figure 2, with numbers on the plots corresponding to protein IDs listed in Table S2. In all volcano plots presented in the main text statistical significance was determined using FDR‐adjusted *p*‐values (Benjamini–Hochberg correction). For Figure S3, unadjusted *p*‐values are shown, as none of the proteins in these comparisons passed the FDR threshold for multiple testing correction.

#### 3.1.1 Up‐regulated protein expression following the initial training session (T1)

Immediately post‐exercise (p1 vs. p0), increases centred on alarmins/S100‐family and cytoskeleton/energy metabolism proteins such as prothymosin‐α (PTMA; *q* = 9.8 × 10^−8^; FC = 1.73), S100A8 (*q* = 2.47 × 10^−15^; FC = 1.71), S100A9 (*q* = 2.07 × 10^−15^; FC = 1.60), phosphoglycerate kinase 1 (PGK1; *q* = 1.91 × 10^−5^; FC = 1.58), decorin (DCN; *q* = 1.94 × 10^−9^; FC = 1.58), peptidyl‐prolyl *cis*–*trans* isomerase (PPIA; *q* = 2.07 × 10^−15^; FC = 1.50), glucose‐6‐phosphate dehydrogenase (G6PD; *q* = 5.55 × 10^−5^; FC = 1.46), S100A12 (*q* = 2.38 × 10^−8^; FC = 1.44) and cofilin‐1 (CFL1; *q* = 2.24 × 10^−10^; FC = 1.44). Full lists are in Table S2.

At 30 min (p2 vs. p0, *n* = 59), elevations largely persisted or strengthened for S100A8 (*q* = 2.45 × 10^−7^; FC = 1.76), S100A9 (*q* = 1.23 × 10^−7^; FC = 1.73), LCN9 (*q* = 5.83 × 10^−8^; FC = 1.60), nucleobindin‐1 (*q* = 1.18 × 10^−10^; FC = 1.56), S100A12 (*q* = 1.07 × 10^−4^; FC = 1.54), PGK1 (*q* = 3.54 × 10^−4^; FC = 1.46), thymosin‐β4 (TMSB4; *q* = 1.18 × 10^−8^; FC = 1.46), tropomyosin‐α4 (*q* = 1.08 × 10^−6^; FC = 1.45), PPIA (*q* = 1.36 × 10^−8^; FC = 1.44) and adenosine kinase (ADK; *q* = 2.7 × 10^−4^; FC = 1.33).

Analysis revealed distinct expression patterns between p1 and p2, with eight proteins exhibiting significant up‐regulation and six proteins displaying significant down‐regulation at p1 relative to p2. Proteins up‐regulated at p1 included DCN (*q* = 3.14 × 10^−6^; FC = 1.77), secretogranin‐1 (CHGB; *q* = 1.33 × 10^−2^; FC = 1.59), serglycin (SRGN; *q* = 1.30 × 10^−4^; FC = 1.47), PTMA (*q* = 3.52 × 10^−2^; FC = 1.35), CAP1 (*q* = 4.54 × 10^−2^; FC = 1.26) and haemoglobin subunits (HBB *q* = 1.33 × 10^−2^; FC = 1.22; HBA *q* = 1.51 × 10^−2^; FC = 1.21). The p2 > p1 changes were an alcohol dehydrogenase E chain (*q* = 2.61 × 10^−2^; FC = 1.22), peptidyl‐prolyl *cis*–*trans* isomerase B (PPIB; *q* = 7.0 × 10^−5^; FC = 1.37), transgelin (TAGLN; *q* = 4.1 × 10^−2^; FC = 1.22), betaine homocysteine *S*‐methyltransferase (*q* = 2.61 × 10^−2^; FC = 1.28), galectin (*q* = 1.51 × 10^−2^; FC = 1.39), nucleobindin‐1 (NUCB1, *q* = 8.47 × 10^−9^; FC = 1.56).

**FIGURE 3:**
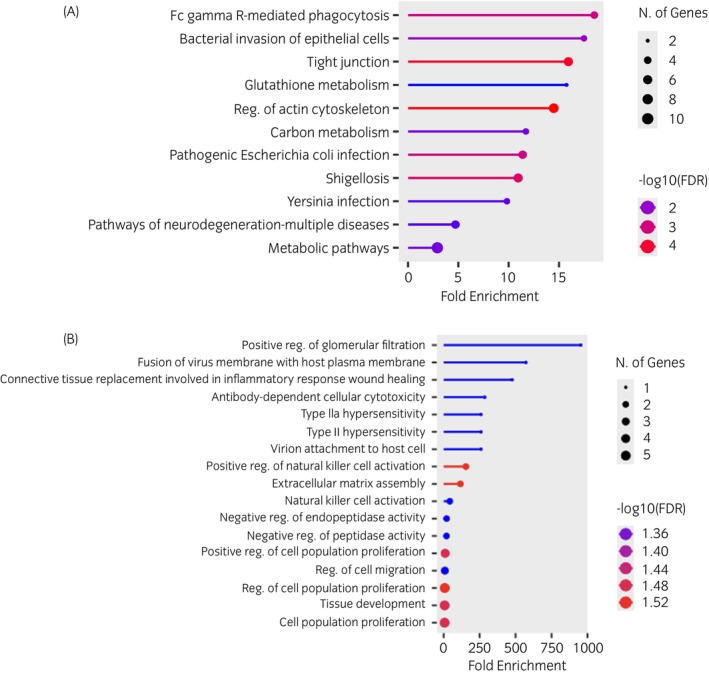
Functional analyses of proteins with altered expression after the initial training session (T1). KEGG pathway enrichment for up‐regulated (A) and down‐regulated proteins (B) (ShinyGO v0.82; species background *Homo sapiens*). Bubble size indicates the number of proteins mapped to each pathway; colour represents statistical significance as −log10(FDR). FDR, false discovery rate.

**FIGURE 4:**
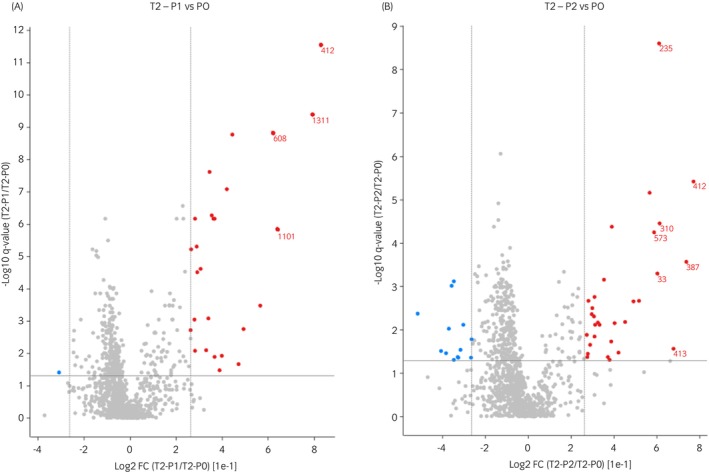
Volcano plot for the mid‐season training session (T2). Protein numbers on the plot indicate IDs of proteins with |FC| > 1.5, corresponding to the identifiers listed in Table S2. (A) Up‐regulated proteins (post‐exercise vs. pre‐exercise) include S100A8 (ID 412), S100A9 (ID 608), S100A12 (ID 1101) and DCN (ID 1311), indicating sustained immune activation and extracellular‐matrix remodelling under continued training adaptation. (B) Up‐regulated proteins (post‐recovery vs. pre‐exercise) include S100A8 (ID 412), NUCB1 (ID 235), LCN9 (ID 310), PGK1 (ID 413) and detoxification‐related enzymes GST (ID 573), GSTA3 (ID 33) and ADH1E (ID 378), suggesting activation of antioxidant and metabolic recovery pathways. Blue dots represent down‐regulated proteins. FC, fold change; NUCB1, nucleobindin‐1; PGK1, phosphoglycerate kinase 1.

Up‐regulated at time point p1 proteins formed a robust PPI network meaning statistically non‐random, high‐confidence connectivity in STRING (Figure S4A); hubs α‐actinin‐1 (ACTN1), CFL1, non‐muscle myosin heavy chain IIA, Myosin‐9 (MYH9), valosin‐containing protein, p97 (VCP) and superoxide dismutase 1 (SOD1) implicated cytoskeletal remodelling, proteostasis and redox defense. KEGG enrichment (Figure 3A)^19^ highlighted FcγR‐mediated phagocytosis, bacterial invasion of epithelial cells, tight junction and actin cytoskeleton regulation, with functional clustering. GO Biological Process terms (Figure 4B) emphasised neutrophil/immune activation, oxidative‐stress response and cytoskeletal control, a profile consistent with acute exercise‐induced remodelling.

#### 3.1.2 Down‐regulated protein expression following the initial training session (T1)

Down‐shifts were fewer and of smaller magnitude. At p1 versus p0, examples included LY75 (*q* = 2.52 × 10^−3^; FC = −1.32), TIMP1 (*q* = 1.70 × 10^−2^; FC = −1.29), EFEMp2 (*q* = 3.92 × 10^−4^; FC = −1.27), PLA1A (*q* = 4.44 × 10^−10^; FC = −1.25), FCGR3A (*q* = 3.06 × 10^−3^; FC = −1.24) and GAS6 (*q* = 2.55 × 10^−2^; FC = −1.23) (Table S2).

Proteins down‐regulated at p2 versus p0 included CD34 molecule (CD34, *q* = 4.55 × 10^−3^; FC = −1.26), gelsolin (*q* = 2.7 × 10^−2^; FC = −1.25), low affinity immunoglobulin gamma Fc region receptor III‐A (LOC100051526; *q* = 1.5 × 10^−3^; FC = −1.31), GAS6 (*q* = 3.71 × 10^−2^; FC = −1.26), integrin alpha‐1 (*q* = 4.0 × 10^−4^; FC = −1.25), chymotrypsin C (*q* = 3.6 × 10^−2^; FC = −1.22), sphingomyelin phosphodiesterase 1 (*q* = 9.92 × 10^−3^; FC = −1.21), carboxylic ester hydrolase 2 (*q* = 3.24 × 10^−2^; FC = −1.23), integrin alpha‐11 (*q* = 1.98 × 10^−4^; FC = −1.25), cystatin C (*q* = 2.47 × 10^−3^; FC = −1.22), integrin subunit alpha 5 (*q* = 2.55 × 10^−2^; FC = −1.25), exostosin‐1 (*q* = 9.42 × 10^−3^; FC = −1.22), activating transcription factor 6 beta (*q* = 6.25 × 10^−3^; FC = −1.23), semaphorin‐6D (*q* = 1.32 × 10^−3^; FC = −1.25) and Ig‐like domain‐containing proteins (*q* = 3.03 × 10^−2^; FC = −1.60).

STRING analysis of the down‐regulated set showed limited direct connectivity (Figure S4C). After adding functional neighbours (STRING ‘More’), implicated pathways included stress/endoplasmic reticulum (ER)‐signalling (ADPRH, CIPC, ARL6IP6), immune/cytokine signalling (FCGR3A, IL13RA1), extracellular matrix (ECM)/tissue remodelling (TIMP1, EFEMP2, PODXL), survival/inflammation (GAS6) and Ca^2+^/metabolic adaptation (PEF1). GO terms (Figures 3B and S4D) pointed to immune effector regulation (e.g., antibody‐dependent cellular cytotoxicity, natural killer cells activity), wound healing/cell migration and protease inhibition, suggesting a controlled down‐tuning of inflammatory/remodelling programs during early recovery.

### 3.2 Protein expression changes reflecting adaptation after mid‐season conditioning (T2)

In p1 versus p0, 311 proteins had *q* < 0.05; 27 met |FC| ≥ 1.2. (26 up‐regulated and 1 down‐regulated). In p2 versus p0, 330 met *q* < 0.05; 44 passed |FC| ≥ 1.2 (31 up‐regulated and 13 down‐regulated). In p2 versus p1, eight proteins reached *q* < 0.05, six with |FC| ≥ 1.2 (Table S2). The differential protein abundance relative to baseline for the T2 session has been presented on volcano plots (Figure 4), with numbers on the plots corresponding to protein IDs listed in Table S2.

#### 3.2.1 Up‐regulated protein expression following the mid‐season conditioning (T2)

The most prominently up‐regulated proteins were S100A8 (*q*‐value = 2.84 × 10^−12^; FC = 1.78), DCN (*q*‐value = 3.96 × 10^−10^; FC = 1.73), S100A12 (*q*‐value = 1.43 × 10^−6^; FC = 1.56) and S100A9 (*q*‐value = 1.50 × 10^−9^; FC = 1.54). Additional increases included PPIA (*q* = 1.69 × 10^−9^; FC = 1.36), TMSB4 (*q* = 8.29 × 10^−8^; FC = 1.34), G6PD (*q* = 6.62 × 10^−7^; FC = 1.29), GSTA3 (*q* = 5.28 × 10^−7^; FC = 1.28), LCN9 (*q* = 6.62 × 10^−7^; FC = 1.29) and PGK1 (*q* = 1.76 × 10^−3^; FC = 1.41).

In p2 versus p0, the most strongly increased proteins included NUCB1 (*q*‐value = 2.5 × 10^−9^; FC = 1.53), S100A8 (*q* = 3.8 × 10^−6^; FC = 1.71), S100A9 (*q* = 6.95 × 10^−6^; FC = 1.48), ADH1E (*q* = 2.68 × 10^−4^; FC = 1.67), LCN9 (*q* = 3.48 × 10^−5^; FC = 1.53), GST (*q* = 5.62 × 10^−5^; FC = 1.50), GSTA3 (*q* = 5.01 × 10^−4^; FC = 1.52) and S100A12 (*q* = 2.11 × 10^−3^; FC = 1.43). Direct comparison of the two post‐training time points showed a single protein up‐regulated in p1 versus p2 at T2—DCN (*q*‐value = 3.64 × 10^−3^; FC = 1.66).

**FIGURE 5:**
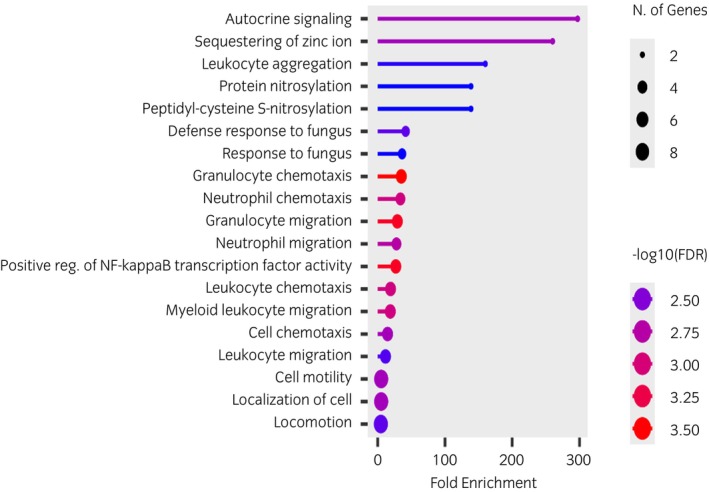
Functional analyses of proteins up‐regulated after the mid‐season training session (T2). KEGG pathway enrichment (ShinyGO v0.82; species background Homo sapiens). Bubble size indicates the number of proteins mapped to each term; colour encodes statistical significance as −log10(FDR). FDR, false discovery rate.

The interaction network (Figure S5A) was well structured, with ACTR3, CFL1, ANXA1, S100A8 and S100A16 serving as key hubs involved in cytoskeletal remodelling, calcium signalling and innate immune response. GO terms (Figure 5) enriched for neutrophil chemotaxis/migration, positive nuclear factor kappa B (NF‐κB) regulation, zinc sequestration, autocrine signalling, protein nitrosylation. Hierarchical clustering of these GO terms (Figure S5B) confirmed that the up‐regulated proteins participate in tightly coordinated immune, metabolic and structural adaptation to exercise.

#### 3.2.2 Down‐regulated protein expression following the mid‐season conditioning (T2)

In p1 versus p0, proteomic analysis revealed only CD34 significantly down‐regulated (*q* = 3.93 × 10^−2^; FC = −1.24) (Table S2).

In p2 versus p0, the most prominent decreases were observed for FAT1 (*q* = 7.59 × 10^−4^; FC = −1.27), COL10A1 (*q* = 9.47 × 10^−4^; FC = −1.28), OLFM4 (*q* = 4.19 × 10^−3^; FC = −1.43), APOM (*q* = 7.58 × 10^−3^; FC = −1.23), PLXND1 (*q* = 9.47 × 10^−3^; FC = −1.29), PCSK5 (*q* = 1.66 × 10^−2^; FC = −1.20), GALNT18 (*q* = 2.84 × 10^−2^; FC = −1.25), SDK2 (*q* = 3.06 × 10^−2^; FC = −1.33), GPNMB (*q* = 4.16 × 10^−2^; FC = −1.26), THOp1 (*q* = 4.39 × 10^−2^; FC = −1.25) and EFEM p2 (*q* = 4.39 × 10^−2^; FC = −1.20).

Direct comparison of the two post‐training time points (p1 vs. p2) showed five proteins down‐regulated (i.e., higher in p2) (Table S3). The latter included NUCB1 (*q* = 1.92 × 10^−6^; FC = −1.49), ADH1E (*q* = 3.14 × 10^−3^; FC = −1.44), PPIB (*q* = 2.04 × 10^−2^; FC = −1.33), DHDH (*q* = 3.39 × 10^−2^; FC = −1.29) and GLUD1 (*q* = 2.81 × 10^−2^; FC = −1.25).

### 3.3 Proteomic response to maximal physical effort: Race‐phase session

**FIGURE 6:**
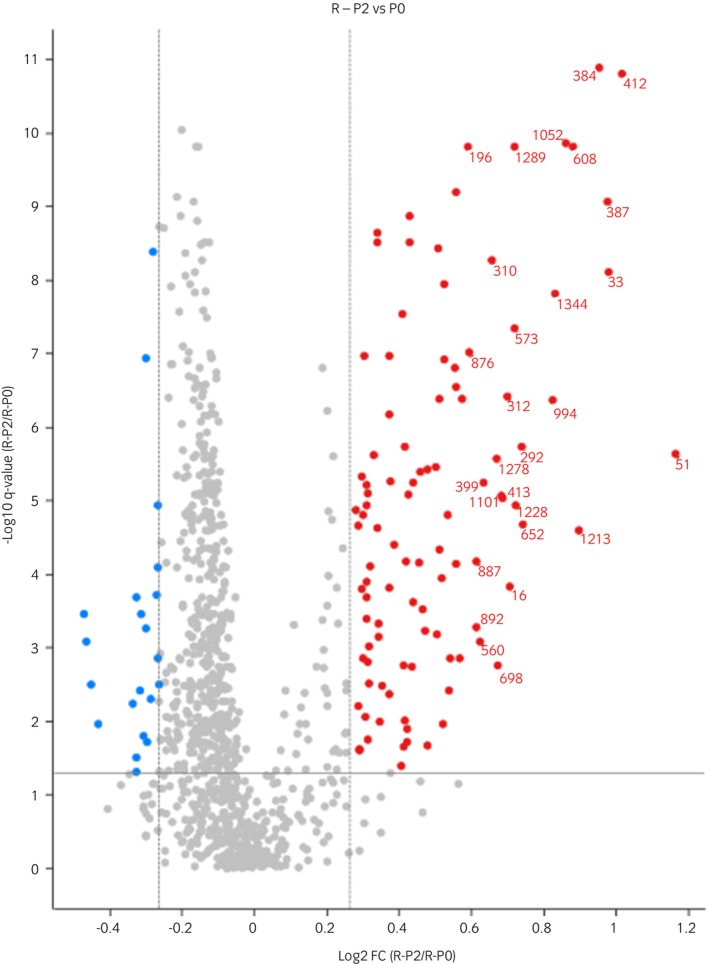
Volcano plot for the race session (R) (post‐recovery vs. pre‐exercise). Protein numbers on the plot indicate IDs of proteins with |FC| > 1.5, corresponding to the identifiers listed in Table S2. Marked up‐regulation of S100A12 (ID 1101), G6PD (ID 1278), PGK1 (ID 413), GSTs (ID 573), ADH1E (ID 387), LCN9 (ID 310), AHSP (ID 892) and related proteins reflects intense erythrocyte turnover, oxidative stress and redox buffering during maximal exertion. Blue dots represent down‐regulated proteins. AHSP, alpha hemoglobin stabilizing protein; G6PD, glucose‐6‐phosphate dehydrogenase; PGK1, phosphoglycerate kinase 1.

In p2 versus p0, 604 proteins reached *q* ≤ 0.05; 103 were up‐regulated and 23 down‐regulated at |FC| ≥ 1.2 (Table S2). Differential protein abundance for the R session has been presented at Figure 6.

#### 3.3.1 Up‐regulated protein expression following the race‐phase session

Maximal effort amplified immune/redox, glycolytic and cytoskeletal programs. Notable increases included PTMA (*q*‐value = 2.33 × 10^−6^; FC = 2.24), S100A8 (*q*‐value = 1.56 × 10^−11^; FC = 2.02), GSTA3 (*q*‐value = 7.88 × 10^−9^; FC = 1.97), ADH1E (*q*‐value = 8.59 × 10^−10^; FC = 1.97), TMSB4 (*q*‐value = 1.3 × 10^−11^; FC = 1.94), DHDH (*q*‐value = 2.51 × 10^−5^; FC = 1.86), S100A9 (*q*‐value = 1.51 × 10^−10^; FC = 1.84), PPIA (*q*‐value = 1.35 × 10^−10^; FC = 1.82), S100G (*q*‐value = 1.52 × 10^−8^; FC = 1.78) and DDT (*q*‐value = 4.23 × 10^−7^; FC = 1.77). Additional significant increases included S100A12 (*q*‐value = 8.66 × 10^−6^; FC = 1.61), NUCB1 (*q*‐value = 3.09 × 10^−9^; FC = 1.35) and DCN (*q*‐value = 1.26 × 10^−4^; FC = 1.24).

**FIGURE 7:**
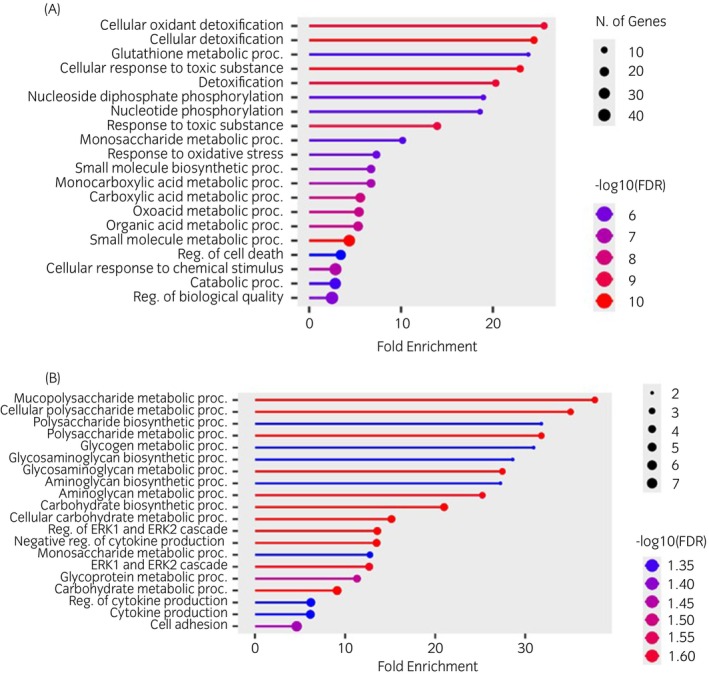
Functional analyses of proteins with altered expression after the race session (R). KEGG pathway enrichment for up‐regulated (A) and down‐regulated proteins (B) (ShinyGO v0.82; species background *Homo sapiens*). Bubble size indicates the number of proteins mapped to each pathway; colour represents statistical significance as −log10(FDR).

STRING revealed a dense network with multiple central hubs (Figure S6A): glycolysis triosephosphate isomerase 1 (TPI1), PGK1, glucose‐6‐phosphate isomerase (GPI), proteostasis‐related (ubiquitin C [UBC], vasolin‐containg protein [VCP]), antioxidant SOD1, cytoskeletal hubs (ACTN1, CFL1) and strong inflammatory ANXA1/S100A8. KEGG enrichment (Figure 7A) highlighted glycolysis/gluconeogenesis, the pentose phosphate pathway, fructose/mannose, nitrogen/arginine metabolism, glutathione metabolism, carbon metabolism, amino acid biosynthesis, drug metabolism‐CYP, peroxisome proliferator–activated receptor signalling, longevity regulation, FcγR phagocytosis. GO terms (Figure S6B) further emphasised oxidative stress defense and oxidant detoxification, glutathione pathways and broad nucleotide/monosaccharide metabolism. The hierarchical GO cluster tree (Figure S6C) revealed convergence of biological processes involved in antioxidant protection, cellular metabolism and inflammation resolution.

#### 3.3.2 Down‐regulated protein expression following the race‐phase session

The most prominent decreases were PCDH18 (*q*‐value = 3.46 × 10^−4^; FC = −1.39), IGF1 (*q*‐value = 8.21 × 10^−4^; FC = −1.38), EFEMp2 (*q*‐value = 3.20 × 10^−3^; FC = −1.37), TRAV16 (*q*‐value = 1.11 × 10^−2^; FC = −1.35), GPNMB (*q*‐value = 5.69 × 10^−3^; FC = −1.26), IL13RA1 (*q*‐value = 2.04 × 10^−4^; FC = −1.25), LRRN4 (*q*‐value = 4.84 × 10^−2^; FC = −1.25), MYH1 (*q*‐value = 3.87 × 10^−3^; FC = −1.25) and EXOC3L4 (*q*‐value = 3.47 × 10^−4^; FC = −1.24) pointing to modulation of ECM/secretory and immune‐regulatory processes (Table S2).

GO enrichment (Figure 7B) emphasised proteoglycan metabolism, peptidyl‐lysine oxidation, sulphotransferase activity, polysaccharide/GAG biosynthesis and hints of satellite‐cell regulation. Subcellular localisation analysis indicated enrichment in the Golgi apparatus and extracellular space, consistent with the secretory or ECM‐related roles of these proteins. Hierarchical clustering revealed tight functional links between polysaccharide metabolism, glycosaminoglycan biosynthesis and cytokine regulation (Figure S6D). STRING mapping of the down‐regulated set revealed limited direct PPI connectivity, as expected for secreted/ECM proteins; however, the existing edges and enriched terms converged on extracellular‐matrix organisation and regulatory signalling (Figure S6C). STRING mapping of the down‐regulated set revealed limited direct PPI connectivity, as expected for secreted/ECM proteins; however, the existing edges and enriched terms converged on extracellular‐matrix organisation and regulatory signalling.

### 3.4 Proteins with altered expression patterns – cross‐session comparisons

No proteins met the prespecified FDR criterion (*q* ≤ 0.05; |FC| ≥ 1.20) in this section; we therefore refrain from claiming statistical significance. Nominal *p*‐values reported below are provided solely to describe patterns and to guide interpretation alongside effect sizes (|FC|). Volcano plot analysis revealed distinct session‐ and time‐dependent differences in post‐exercise protein abundance (Figure S3). At p1, only limited changes were detected between T2 and T1 (Figure S3A), whereas more pronounced differences emerged at p2 (Figure S3B). Stronger shifts were observed in comparisons involving the race phase, with multiple proteins differentially abundant (DA) in R versus T1 (Figure S3C) and R versus T2 (Figure S3D) at p2.

Comparing the two training stages (median FC T1–p1 vs. T2–p1; positive FC = higher after T1; negative FC = higher after T2), no proteins passed FDR (*q* < 0.05). However, two proteins met nominal *p* < 0.05 (|FC| ≥ 1.20): PCTP (FC = 1.27; nominal *p* = 7.59 × 10^−5^) and SOD1 (FC = 1.20; *p* = 2.17 × 10^−3^). Full lists are provided in Table S3.

Comparing the two training stages (median FC T1–p2 vs. T2–p2), no proteins passed FDR (*q* < 0.05). However, five proteins met nominal *p* < 0.05 (|FC| ≥ 1.20); see Table S3. Relative to T1, R (p2) exhibited higher abundance of multiple markers of oxygen transport, cytoskeletal/actin dynamics, redox/homeostatic responses and lipid/fatty‐acid handling. Multiple proteins reached statistical significance after FDR control; counts and per‐protein statistics are reported in Table S3.

At 30‐min post‐exercise (p2), 85 proteins reached *q* ≤ 0.05 in R versus T2, of which 56 met |FC| ≥ 1.20 (50 higher in R; 6 higher in T2) see Table S3 for the complete list.

**FIGURE 8:**
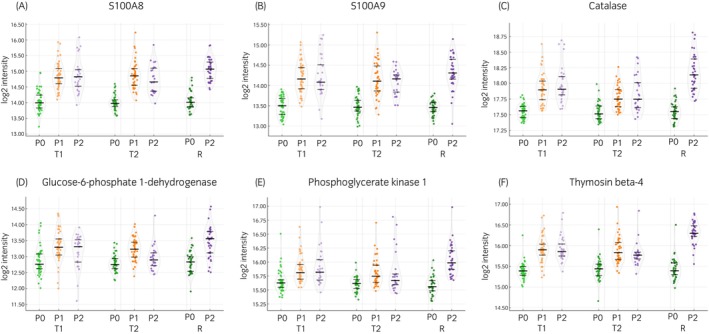
Violin plots showing the distribution of log₂ intensities for six plasma proteins across sessions and sampling timepoints. (A) S100A8, (B) S100A9, (C) Catalase, (D) glucose‐6‐phosphate 1‐dehydrogenase (G6PD), (E) phosphoglycerate kinase 1 (PGK1), (F) thymosin β4. The *X*‐axis is arranged in session blocks—T1 (initial—first training), T2 (mid‐season training—second training) and R (race‐phase) with timepoints inside each block: p0 (pre‐exercise), p1 (immediately post‐exercise), p2 (30 min post‐exercise). Each violin depicts the full distribution; the thick horizontal line marks the median, thin bars indicate Q1 and Q3, and dots represent individual samples. Dot colours encode timepoints: green p0, orange p1, purple p2. The *Y*‐axis shows log_2_ intensity. This visualisation enables side‐by‐side comparison of central tendency, dispersion and distribution shape within and between sessions and timepoints, highlighting exercise‐related shifts and potential features such as skewness or multimodality.

Based on proteins consistently modulated across sessions, we propose a preliminary candidate panel for monitoring training adaptation and peak load (Table 1). The panel integrates immune/stress sensors (S100A8, S100A9, TMSB4), antioxidant enzymes (SOD1, catalase, GST/G6PD), metabolic markers (PGK1, G6PD, lipocalins) and structural modulators (DCN, CFL1). Median FC values from within‐ and between‐session contrasts are reported to illustrate effect sizes. These targets warrant validation by ELISA or LC–MRM/PRM in larger, independent cohorts. Six example proteins with the largest session‐wide shifts are summarised in Figure 8 and Table S4.

### 3.5 Key results

Initial training (T1) yielded 63 DA proteins at p1 versus p0 (54↑/9↓) and 75 at p2 versus p0 (59↑/16↓); mid‐season conditioning (T2) showed 27 and 44, respectively; race‐phase (R) produced 126 (103↑/23↓) at p2 versus p0. R versus T2 (p2) added 56 features (50↑ in R/6↑ in T2). Across all sessions, a consistent sentinel subset emerged: S100A8, S100A9, S100A12, TMSB4, PTMA, PGK1, G6PD, SOD1, CAT, CFL1 and DCN, representing coordinated axes of innate immune activation, redox and energetic support and cytoskeletal–ECM remodelling.

## 4 DISCUSSION

### 4.1 Early proteomic response to initial training session (T1)

The first training session elicited a rapid, broad proteomic response spanning innate immunity, energy/redox metabolism and cytoskeletal—matrix remodelling. Members of the S100 family—S100A8/S100A9 (calprotectin) with S100A12—dominated the early signal (the top FC/q hits at p1 versus p0, sustained at p2 versus p0). This pattern is consistent with neutrophil priming and alarmin‐mediated signalling triggered by exercise‐induced microtrauma (i.e., DAMP activity^20^, ^21^). It also corresponds to the pronounced post‐exercise induction of S100 genes reported in horses^22^ and may reflect compartmental dynamics, such as sweat‐associated protein loss immediately following endurance work.^23^ Additionally, it parallels contexts in which elevated S100 levels are observed in disease states, including transport‐associated respiratory disease and colic in horses.^24^, ^25^, ^26^ Functionally, KEGG enrichment pointed to FcγR‐mediated phagocytosis, bacterial invasion of epithelial cells and tight‐junction signalling, while GO terms emphasised neutrophil activation, chemotaxis and leukocyte migration. Together with the actin‐centred hubs (ACTN1, CFL1, MYH9) in the PPI map, these pathways support a picture of cytoskeleton‐dependent immune cell mobilisation and phagocytic competence early after load initiation.

Metabolically, the data indicate acute reprogramming towards glycolysis and nicotinamide adenine dinucleotide phosphate (NADPH) support: increases in PGK1 and G6PD (pentose phosphate pathway) suggest both higher ATP demand and redox poise maintenance via NADPH provision. KEGG/GO enrichment corroborated this energetic/redox axis, given the centrality of pentose phosphate pathway (PPP) to reactive oxigen species (ROS) detoxification in working muscle.^27^, ^28^, ^29^

Early lipid‐associated protein changes were present but more modest. Increases in lipocalin (e.g., LCN9; sustained at p2P2) are compatible with training‐induced lipid remodelling.^30^ By contrast with endurance studies reporting ApoA‐IV/ApoE rises and ApoC‐II/ApoC‐III decreases,^10^ all four apolipoproteins were detectable here but sub‐threshold at T1, which likely reflects differences in exercise modality, sampling timing (immediate/30 min) and training status. Longer‐term plasticity of lipid transport seen in age‐stratified cohorts^11^ may also explain the relatively small acute T1 effects in our plasma proteome.

Markers of cytoskeletal turnover and tissue repair were also prominent. TMSB4, PTMA and CFL1 increased, while ACTN1 appeared as a central hub in the interaction network. Together with an early rise in DCN, these signals point to actin dynamics and ECM/tendon micro‐remodelling that accompany load initiation.^31^, ^32^, ^33^, ^34^ The p1 > p2 pattern for DCN and secretory granule components (CHGB, SRGN) versus p2 > p1 increases in PPIB, NUCB1, TAGLN suggests a temporal hand‐off from rapid injury/traction signalling to protein folding/trafficking and cytoskeletal consolidation within the first 30 min. The ECM‐related signal, including the rapid response of DCN in p1, may also reflect peritendinous remodelling processes associated with tenocyte traction signalling.

Regarding antioxidant defenses, T1 dataset emphasises redox network activation rather than large, uniform increases in canonical ROS‐scavenging enzymes at the immediate time points. SOD1 emerged as a network hub (with PPP support via G6PD), yet literature on acute post‐exercise SOD/catalase activity in equine plasma is mixed, including reports of no significant immediate change immediately following an endurance race.^35^, ^36^ This variability likely reflects compartmentalisation (plasma vs. tissue), timing and exercise type, and it is consistent with our observation that redox homeostasis at T1 is supported by metabolic routing (PPP/NADPH) alongside selective protein‐level adjustments.

Finally, the down‐regulated set at T1 was smaller and less connected. Decreases in proteins related to immune receptors (e.g., LY75, FCGR3A) and ECM modulators (e.g., TIMP1, EFEMp2) are compatible with a controlled dampening of inflammatory/remodelling drivers during early recovery, a pattern that fits with the GO terms enriched for immune effector regulation and wound‐healing/cell migration and protease inhibition.^37^ STRING expansion suggested links to cytokine signalling nodes (e.g., IL13RA1) and ER‐stress/redox adaptors, supporting the notion that pro‐inflammatory activation is counter‐balanced by negative‐feedback and tissue‐protective programmes within the first hour.

Collectively, T1 couples alarmin‐driven innate immune activation with energy/redox rerouting and cytoskeletal–matrix priming, establishing a pro‐adaptation molecular milieu that matches the volcano/network/enrichment architecture and the targeted protein shifts.

### 4.2 Refined proteomic profile during mid‐season training conditioning (T2)

By mid‐season, the plasma proteome shifted from the broad, acute reactivity seen at T1 towards a more focused and stabilised adaptation. The S100 axis remained a defining feature—S100A8/A9/A12 increased at both p1 and p2, together with DCN and CFL1. STRING hubs (ACTR3, CFL1, ANXA1, S100A8, S100A16) and GO terms (neutrophil chemotaxis; leukocyte/granulocyte migration; positive NF‐κB signalling; zinc‐ion sequestration; autocrine signalling; protein nitrosylation) indicate preserved innate readiness (migration/adhesion, NF‐κB priming) balanced with resolution‐biased control (e.g., ANXA1 as a pro‐resolving mediator; S100‐driven zinc sequestration as ‘nutritional immunity’). In practical terms, this reads as fewer proteins altered, but those that do change converge on coordinated immune trafficking and barrier/adhesion control rather than diffuse activation.^38^, ^39^

Compared with T1, T2 maintained increases in glycolysis (PGK1) and PPP support (G6PD) but showed proportionally stronger signals in detox/glutathione pathways: GSTA3/GST rose (p1 and p2) and ADH1E and DHDH emerged as carbonyl/xenobiotic‐handling nodes.^27^, ^28^, ^40^ Together with NUCB1 and LCN9, these point to a more selective antioxidant/ligand‐handling program—consistent with training adaptation that prioritises ROS buffering and metabolic by‐product clearance over the broader enzyme up‐shifts observed at T1. On the structural side, DCN remained prominently up at p1 (and the sole p1 > p2 protein at T2), suggesting an immediate ECM/tenocyte traction signal, whereas p2 favoured protein‐folding/secretory and metabolic consolidation (NUCB1, PPIB, ADH1E, DHDH, GLUD1 higher at p2).^41^, ^42^, ^43^

The down‐set at T2 was minimal at p1 versus p0 (only CD34) and selective at p2 versus p0 (e.g., FAT1, COL10A1, OLFM4, APOM, PLXND1, PCSK5, GALNT18, SDK2, GPNMB, THOp1, EFEMp2). Functionally, the pattern is compatible with controlled attenuation of adhesion/ECM remodelling and glycoprotein/secretory processing, that is, resolution‐aligned pruning rather than wholesale suppression. The transient decrease in CD34 may reflect short‐term endothelial/progenitor marker dynamics with acute loading, but we interpret this cautiously given the single‐protein signal and plasma context.

### 4.3 Intense proteomic response to race‐phase effort (R)

The race‐phase (R) elicited the most extensive and integrated proteomic shift of the study. The S100 axis intensified, with S100A8 (FC = 2.02), S100A9 (1.84), S100A12 (1.61) and S100G (1.78) among the most elevated proteins, accompanied by ANXA1 as an inflammatory/pro‐resolving hub in the network. Relative to the training sessions (standardised to p2 vs. p0), S100A8 and S100A9 reached their maxima at R (T1: 1.76/1.73; T2: 1.71/1.48), together with KEGG enrichment for FcγR‐mediated phagocytosis, indicating strong neutrophil activation and phagocytic readiness. However, it should be interpreted at the plasma proteome level, where the increase in neutrophil‐derived proteins likely reflects cell activation and relocalisation rather than a direct increase in phagocytic activity in vivo. While cellular leakage/haemolysis can contribute to apparent increases in circulating granulocyte proteins,^26^ the STRING and GO architecture supports a genuine innate‐immune mobilisation coherent with maximal exertion. Network analysis revealed a glycolytic module (TPI1, PGK1, GPI) and PPP enrichment consistent with high ATP and NADPH demand. Detoxification/antioxidant capacity was markedly reinforced: GSTA3 (1.97) and ADH1E (1.97), together with DHDH (1.86) and PPIA (1.82), point to glutathione‐dependent conjugation and carbonyl/xenobiotic clearance, in line with GO terms for oxidant detoxification and response to toxic compounds. Prior work indicates training‐induced modulation of glutathione‐linked enzymes during 2 years of high‐intensity training in horses^11^; the GST surge at R is congruent with acute ROS neutralisation at peak load.^27^, ^28^, ^44^

Cytoskeletal control intensified (CFL1, ACTN1 hubs), while VCP and UBC signalled proteostasis/ubiquitin‐dependent turnover—a canonical response to proteotoxic stress.^45^ Proteins linked to regeneration and actin dynamics rose sharply—PTMA (2.24) and TMSB4 (1.94)—consistent with rapid structural adaptation and early repair signalling. Together, these modules align with the functional integration seen in KEGG clustering, where immunity, metabolism and stress‐response pathways converge.

The down‐set involved ECM/secretory and signalling nodes, including IGF1 (−1.38), EFEMp2 (−1.37) and PCDH18 (−1.39). GO highlights proteoglycan metabolism, peptidyl‐lysine oxidation and sulphotransferase activity, together with terms implicating satellite‐cell regulation. This pattern suggests a transient suppression/re‐prioritisation of matrix/growth signalling post‐race, potentially reallocating resources towards detoxification, redox control and cytoskeletal resilience.^46^, ^47^, ^48^ However, the observed decreases in ECM‐ and IGF1‐related signals in R–p2 may also reflect a short‐term re‐allocation of resources away from matrix maintenance, rather than unequivocal proteolysis. This interpretation requires further validation using dedicated ECM marker panels, such as collagen pro‐peptides and assays of MMP activity.

Overall, R captures a peak‐workload molecular signature: alarmin‐driven innate activation, high‐flux energy/PPP support, glutathione‐centred detox, proteostasis and rapid cytoskeletal–repair cues. As a cautious take‐home plasma marker panel for ‘peak load’, PTMA, TMSB4, S100A8/A9, GSTA3 and ADH1E emerge as candidates—pending independent validation and strict control for haemolysis. The dense STRING network and pathway clustering emphasise that these processes are co‐regulated, not merely co‐occurring.

### 4.4 Integrated comparison and proposed biomarkers of training adaptation

Within‐session contrasts show a transient, T1‐dominant erythrocyte/oxygen‐transport and redox imprint immediately post‐exercise (p1), shifting at T2—particularly at recovery (p2)—towards ECM/immune/coagulation features. This trajectory aligns with a move from broad early reactivity to focused adaptation later in training^8^, ^11^ and is consistent with the concept of trained tolerance—attenuated breadth with preserved specificity.^49^

Following the first session, we observed a rapid inflammatory–redox signature consistent with acute systemic stress. Particularly S100A8/A9 rose early as sensors of immune activation and physiological strain, accompanied by TMSB4 and PTMA, suggesting onset of cytoprotective/angiogenic programs that also feature during racing. As S100G is typically an intracellular protein, its presence in plasma most likely reflects release from cells (muscle, intestine, leukocytes) and/or microdisruptions of cellular membranes. It should also be noted that there is a potential risk of co‐occurrence with an RBC‐related signature. Increased PGK1 and G6PD point to glycolysis/PPP‐driven metabolic reprogramming with enhanced redox buffering, while antioxidant defenses (e.g., SOD1, catalase) support oxidative‐stress control^20^, ^21^, ^27^, ^28^, ^29^, ^31^, ^32^, ^33^, ^35^, ^36^ Collectively, S100A8, S100A9, TMSB4, PTMA, PGK1, G6PD, SOD1 and catalase emerge as early‐phase biomarker candidates at T1.

At mid‐season conditioning, the number of significantly altered proteins decreased and the profile narrowed, indicating stabilised responses. Continued up‐regulation of S100A8/A9, DCN and CFL1 indicates modulated inflammatory and structural remodelling, while increases in glutathione S‐transferases, PPIA and NADP‐dependent oxidoreductases support maintained redox control. A notable decrease of CD34 suggests reduced regenerative demand under routine conditioning. Thus, sustained S100A8/A9, GST, DCN, CFL1 and CD34↓ form a parsimonious mid‐season adaptation set. However, the differences observed between T1 and T2 at *p* < 0.05 (uncorrected for FDR) should be considered exploratory findings.

The Race‐Phase elicited the marked up‐regulation of PTMA, S100 family proteins, TMSB4 and key glycolytic/antioxidant enzymes (e.g., GSTA3, CAT, G6PD, ADH1E) indicate high energetic demand with intensified detox/redox control. Concurrent down‐modulation of ECM/adhesion and immune‐receptor modules (e.g., fibulins/EFEMP, collagens, IL‐13Rα1, selected C‐type lectins) suggests short‐term reprioritisation away from remodelling/surveillance during peak output—compatible with potential physiological peak load. However, the present data indicate a peak physiological strain thus the identified markers should be regarded as candidates for monitoring load/fatigue and require clinical validation with overtraining phenotyping.

Across sessions, consistently modulated proteins, S100A8/A9, TMSB4, GSTs, PPIA/NUCB1, DCN, PGK1, G6PD, SOD1, CAT, may represent promising longitudinal indicators of training status/readiness. Lipocalins/cytosolic fatty‐acid‐binding domain proteins show contrast‐dependent shifts, implicating lipid transport during high demand. A preliminary monitoring panel spanning may be proposed: immune/stress (S100A8, S100A9, TMSB4), antioxidant (SOD1, GST, CAT), energy metabolism (PGK1, G6PD, lipocalins) and structural remodelling (DCN, ARP3, CFL1), with TIMP1, IL‐13Rα1 and selected ECM components as potential peak load flags (down‐modulation). Practical deployment requires targeted validation (ELISA or targeted MS), larger cohorts and longitudinal sampling to resolve effect size versus kinetics, platform variance and environmental/individual modifiers.

An additional layer of interpretation may involve extracellular vesicles (EVs), which have been increasingly recognised as carriers of membrane‐associated proteins released after exercise. In human studies, EVs have also been linked to transient changes in endothelial progenitor–like signals, such as CD34, providing a possible explanation for the temporary shifts observed in our dataset.^50^, ^51^ Considering EV‐mediated release in sport horses may therefore offer a mechanistic framework that connects cellular activation and remodelling with the appearance of circulating protein markers. Future work integrating EV profiling with plasma proteomics could validate this hypothesis.

### 4.5 Main limitations

This study has several limitations. The breed distribution was unbalanced (Arabians predominated) and not all horses completed every sampling. While this reduces generalisability, the within‐subject design, consistent training, and paired statistics mitigate confounding, and exploratory checks did not indicate age/sex bias. Immediate post‐race sampling (R‐p1) was not feasible, so inter‐session effects rely on R‐p2 versus T1/T2‐p2; because p1–p2 differences were modest at T1 and T2, R‐p2 likely captures the dominant race signature. Strong increases in hemoglobin subunit alpha/hemoglobin subunit beta (HBA/HBB), carbonic anhydrase 1/carbonic anhydrase 2, alpha hemoglobin stabilizing protein and biliverdin reductase B, especially in R versus T2, are consistent with erythrocyte admixture/haemolysis; exercise‐induced haemolysis is well documented,^52^, ^53^ and visibly haemolysed samples were excluded as recommended,^54^ though subclinical haemolysis may itself carry information on training load.^52^, ^53^ TMT‐based quantification can attenuate FCs (ratio compression) without altering directionality, so targeted assays (ELISA, MRM, PRM) may show larger effects.^54^, ^55^ We did not perform targeted verification (PRM/SRM) or ELISA in this cohort, which is an explicit limitation of a discovery‐phase study. Given expected TMT ratio compression, effect sizes reported here are conservative and require targeted follow‐up in independent samples. The availability of equine‐reactive assays for selected proteins (e.g., calprotectin, DCN) supports feasibility; however, antibody cross‐reactivity and matrix effects warrant careful validation. Signals involving latherin (Equc1) warrant caution: contamination is unlikely with sterile vacutainers, yet as a secreted lipocalin it may contribute to plasma signal; genuine regulation cannot be excluded and requires orthogonal validation.^56^, ^57^ More broadly, the axes we report—innate activation (e.g., S100A8/A9/A12), redox/energetic support and cytoskeletal/ECM remodelling—are not specific to exercise alone, and plasma offers limited tissue‐of‐origin specificity with potential extracellular‐vesicle contributions; establishing physiological and clinical specificity will therefore require independent validation under field conditions, including temporal profiling and interference testing (haemolysis/lipemia, dehydration/haemoconcentration). Finally, although our indication differs, successful plasma‐proteomics applications in equine disease (e.g., EMS) support the robustness and translational potential of blood‐based proteomics.^58^

## 5 CONCLUSIONS

This study reveals distinct proteomic signatures associated with different stages of racehorse training and racing, reflecting immune activation, metabolic shifts, antioxidant defense and tissue remodelling. Early training triggered broad stress responses, while mid‐season adaptation showed more focused regulation. Racing induced the strongest changes, including markers of high metabolic demand and signs of potential physiological peak load.

Key proteins—such as S100A8, S100A9, thymosin β4, PGK1, G6PD and catalase—emerged as potential biomarkers of training response and fatigue (Table 1 and Figure 8). A proposed biomarker panel may support monitoring of performance, recovery, peak physiological strain and trajectories of adaptation. However, these candidates require further validation before clinical application. Given the conserved nature of many stress, immune and metabolic pathways, these findings may also have broader translational relevance for athletic monitoring and fatigue assessment in other species, including humans.

## FUNDING INFORMATION

This research was funded by the National Science Centre, Poland, No. 2021/41/B/NZ7/03548 (O.W.P.), and the publication was (co)financed by the Science Development Fund of the Warsaw University of Life Sciences—SGGW.

## CONFLICT OF INTEREST STATEMENT

The authors declare that the research was conducted in the absence of any commercial or financial relationships that could be construed as a potential conflict of interest.

## AUTHOR CONTRIBUTIONS

**Jowita Grzędzicka:** Conceptualization; investigation; writing – original draft; visualization; writing – review and editing; software; formal analysis; data curation. **Bianka Świderska:** Conceptualization; investigation; writing – review and editing; writing – original draft; visualization; validation; methodology; data curation; software; formal analysis. **Ewa Sitkiewicz:** Investigation; writing – review and editing. **Izabela Dąbrowska:** Writing – review and editing; investigation. **Olga Witkowska‐Piłaszewicz:** Conceptualization; investigation; funding acquisition; writing – original draft; methodology; writing – review and editing; formal analysis; project administration; supervision; resources.

## DATA INTEGRITY STATEMENT

Olga Witkowska‐Piłaszewicz, Jowita Grzędzicka, Bianka Świderska had full access to all the data in the study and take responsibility for the integrity of the data and the accuracy of data analysis.

## ETHICAL ANIMAL RESEARCH

Research ethics committee oversight not currently required by this journal: procedures were performed as part of routine veterinary examinations conducted as part of standard health and performance monitoring.

## INFORMED CONSENT

Explicit informed consent for this study was obtained from the horses' trainer.

## Supporting information


**Figure S1.** Principal component analysis (PCA) before and after batch‐effect removal with ComBat. Points are coloured by TMT set. (A) The uncorrected data, where samples cluster within TMT sets, indicating pronounced batch structure. (B) The data after ComBat normalisation, where TMT‐driven separation is largely eliminated and set‐specific clustering is no longer apparent. Axes display PC1 and PC2; each point represents one sample.

**Figure S2.** Scree plot of principal components. Percentage of total variance explained by the first 19 PCs calculated from the log₂‐transformed, ComBat‐normalised plasma‐proteomics matrix (all sessions and time points combined).

**Figure S3.** Volcano plots comparing post‐exercise protein abundance between sessions. (A) Compares T2 to T1 at p1; (B) compares T2 to T1 at p2; (C) compares R to T1 at p2; (D) compares R to T2 at p2 (T1, initial training; T2, mid‐season training; R, race‐phase; p1, immediately post‐session; p2, 30‐min post‐session). The *x*‐axis shows log2 fold change (FC) and the y axis − log10(FDR *q*‐value). Reference lines indicate statistical significance (horizontal line at 1.30, corresponding to *q*‐value 0.05) and effect size (vertical lines at 0.263, corresponding to 1.2‐fold). Proteins meeting both thresholds are highlighted: red for higher abundance in the first‐named session of each comparison, blue for lower abundance. Each point represents one protein.

**Figure S4.** Functional analyses of proteins with altered expression after the initial training session (T1). (A, B) Up‐regulated proteins; (C, D) down‐regulated proteins. (A) STRING protein–protein interaction (PPI) network for the up‐regulated set (species background *Equus caballus*; paired *p* ≤ .05, *q* ≤ 0.05; median fold change 1.355–2.24). (C) STRING PPI network for the down‐regulated set (species background *Equus caballus*; paired *p* ≤ .05, *q* ≤ 0.05). (B, D) Hierarchical clustering dendrogram of enriched pathways (background Homo sapiens); node size reflects significance (*p*‐value).

**Figure S5.** Functional analyses of proteins up‐regulated after the mid‐season training session (T2). (A) STRING protein–protein interaction network for the up‐regulated set (species background *Equus caballus*; paired *p* ≤ 0.05, *q* ≤ 0.05; median fold change 1.355–2.24). (B) Hierarchical clustering dendrogram of enriched pathways (background Homo sapiens); node size reflects pathway significance (*p*‐value).

**Figure S6.** Functional analyses of proteins with altered expression after the race session (R). (A, B) Up‐regulated proteins; (C, D) down‐regulated proteins. (A) STRING protein–protein interaction (PPI) network for the up‐regulated set (species background *Equus caballus*; paired *p* ≤ .05, *q* ≤ 0.05; median fold change 1.355–2.24). (C) STRING PPI network for the down‐regulated set (species background Homo sapiens; paired p ≤ 0.05, q ≤ 0.05). (B, D) Hierarchical clustering dendrogram of enriched pathways (background Homo sapiens); node size reflects pathway significance (P value).

**Table S1.** Descriptive characteristics of the racehorses enrolled in the study.

**Table S2.** Differentially expressed plasma proteins between pre‐training (T1‐p0, T2‐p0, R‐p0) and post‐training (T1‐p2, T2‐p2, R‐p2) phases in racehorses, with fold changes (FC) and false discovery rate (FDR) adjusted *p*‐values (*q*‐values) for paired comparisons across different time points. Green backgrounds indicate statistical significance (*q*‐value FDR ≤0.05), while shades of blue/red represent the direction and magnitude of the effect (more intense colours = greater absolute FC value).

**Table S3.** Changes in plasma protein concentrations after exercise (p1 and p2) in T1 (initial training 1), T2 (mid‐season training) and R (race‐phase); paired comparisons are shown for T1–T2, T1–R and T2–R. FC (fold change) values are inverted: a negative value indicates a higher protein concentration in the condition on the right side of the comparison, while a positive value indicates a higher concentration in the condition on the left. FDR values after Benjamini–Hochberg correction and the median FC for each comparison are provided. Green backgrounds indicate statistical significance (FDR <0.05), while shades of blue/red represent the direction and magnitude of the effect (more intense colours = greater absolute FC value).

**Table S4.** Summary of candidate protein biomarkers prioritised based on proteomic analysis. The table lists proteins identified as differentially expressed between time points (T1, P2 vs. T), together with prioritisation level (high/medium), number of detection hits, fold change (|FC|), involvement as potential network hubs, predicted subcellular localisation and associated risk assessment. Additional columns include verification of unique peptides, number of peptides detected and percentage of samples passing quality control (≥50%). High‐priority proteins (e.g., S100A9, S100A12, S100A8, G6PD, PPIA, TMSB4, DCN, PGK1, NUCB1) showed multiple detection hits, moderate to strong fold changes and consistent detection across ≥50% of the dataset. Several proteins were localised to secreted compartments (e.g., S100A9, S100A12, S100A8) or intracellular pathways (e.g., G6PD, SOD1, CFL1), suggesting both extracellular and intracellular mechanisms. Medium‐priority proteins (e.g., GLO1, S100G, VCP, FABP1, CAT, ENO1) generally exhibited fewer hits but remained consistently above the detection threshold.

## Data Availability

The mass spectrometry proteomics data have been deposited to the ProteomeXchange Consortium via the PRIDE partner repository (dataset identifier PXD067028, DOI: https://doi.org/10.6019/PXD067028).

## References

[1] Leisson K , Jaakma Ü , Seen T . Adaptation of equine locomotor muscle fiber types to endurance and intensive high speed training. J Equine Vet Sci. 2008;28:395–401. 10.1016/j.jevs.2008.05.007

[2] Rivero J , Hill E . Skeletal muscle adaptations and muscle genomics of performance horses. Vet J. 2016;209:5–13. 10.1016/j.tvjl.2015.11.019 26831154

[3] Wang T , Meng J . Differential metabolomics and cardiac function in trained vs. untrained Yili performance horses. Animals. 2025;15(16):2444. 10.3390/ani15162444 40867773 PMC12382719

[4] Bryan K , McGivney BA , Farries G , McGettigan PA , McGivney CL , Gough KF , et al. Equine skeletal muscle adaptations to exercise and training: evidence of differential regulation of autophagosomal and mitochondrial components. BMC Genomics. 2017;18:595. 10.1186/s12864-017-4007-9 28793853 PMC5551008

[5] Witkowska‐Piłaszewicz O , Malin K , Dąbrowska I , Grzędzicka J , Ostaszewski P , Carter C . Immunology of physical exercise: is *Equus caballus* an appropriate animal model for human athletes? Int J Mol Sci. 2024;25(10):5210. 10.3390/ijms25105210 38791248 PMC11121269

[6] Lee EC , Fragala MS , Kavouras SA , Queen RM , Pryor JL , Casa DJ . Biomarkers in sports and exercise: tracking health, performance, and recovery in athletes. J Strength Cond Res. 2017;31(10):2920–2937. 10.1519/JSC.0000000000002122 28737585 PMC5640004

[7] Ichibangase T , Imai K . Application of fluorogenic derivatization‐liquid chromatography‐tandem mass spectrometric proteome method to skeletal muscle proteins in fast Thoroughbred horses. J Proteome Res. 2009;8(4):2129–2134. 10.1021/pr801004s 19714884

[8] Bouwman FG , van Ginneken MME , Noben JP , Royackers E , de Graaf‐Roelfsema E , Wijnberg ID , et al. Differential expression of equine muscle biopsy proteins during normal training and intensified training in young standardbred horses using proteomics technology. Comp Biochem Physiol D. 2010;5(1):55–64. 10.1016/j.cbd.2009.11.001 20374942

[9] Scoppetta F , Tartaglia M , Renzone G , Avellini L , Gaiti A , Scaloni A , et al. Plasma protein changes in horse after prolonged physical exercise: a proteomic study. J Proteomics. 2012;75(14):4494–4506. 10.1016/j.jprot.2012.04.014 22546489

[10] Gotić J , Špelić L , Kuleš J , Horvatić A , Gelemanović A , Ljubić BB , et al. Proteomic analysis emphasizes the adaptation of energy metabolism in horses during endurance races. BMC Vet Res. 2025;21:67. 10.1186/s12917-025-04518-0 39955578 PMC11830206

[11] Johansson L , Ringmark S , Bergquist J , Skiöldebrand E , Widgren A , Jansson A . A proteomics perspective on 2 years of high‐intensity training in horses: a pilot study. Sci Rep. 2024;14(1):23684. 10.1038/s41598-024-75266-8 39390056 PMC11467344

[12] Amiri Roudbar M , Rosengren MK , Mousavi SF , Fegraeus K , Naboulsi R , Meadows JRS , et al. Effect of an endothelial regulatory module on plasma proteomics in exercising horses. Comp Biochem Physiol D Genomics Proteomics. 2024;52:101265. 10.1016/j.cbd.2024.101265 38906044

[13] Sullivan GM , Feinn R . Using effect size—or why the p value is not enough. J Grad Med Educ. 2012;4(3):279–282. 10.4300/JGME-D-12-00156.1 23997866 PMC3444174

[14] Hughes CS , Moggridge S , Müller T , Sorensen PH , Morin GB , Krijgsveld J . Single‐pot, solid‐phase‐enhanced sample preparation for proteomics experiments. Nat Protoc. 2019;14(1):68–85. 10.1038/s41596-018-0082-x 30464214

[15] Tyanova S , Temu T , Cox J . The MaxQuant computational platform for mass spectrometry‐based shotgun proteomics. Nat Protoc. 2016;11(12):2301–2319. 10.1038/nprot.2016.136 27809316

[16] Tyanova S , Temu T , Sinitcyn P , Carlson A , Hein MY , Geiger T , et al. The Perseus computational platform for comprehensive analysis of (prote)omics data. Nat Methods. 2016;13(9):731–740. 10.1038/nmeth.3901 27348712

[17] FDA‐NIH Biomarker Working Group . In: Food and Drug Administration (US) , editor. BEST (Biomarkers, EndpointS, and other Tools) resource [Internet]. Silver Spring, MD: FDA‐NIH Biomarker Working Group; 2016 [cited 2025 Nov 3]. Available from: http://www.ncbi.nlm.nih.gov/books/NBK326791/ 27010052

[18] Rifai N , Gillette MA , Carr SA . Protein biomarker discovery and validation: the long and uncertain path to clinical utility. Nat Biotechnol. 2006;24(8):971–983. 16900146 10.1038/nbt1235

[19] Kanehisa M , Goto S . KEGG: Kyoto encyclopedia of genes and genomes. Nucleic Acids Res. 2000;28(1):27–30. 10.1093/nar/28.1.27 10592173 PMC102409

[20] Ryckman C , Vandal K , Rouleau P , Talbot M , Tessier PA . Proinflammatory activities of S100 proteins: S100A8, S100A9, and S100A8/A9 induce neutrophil chemotaxis and adhesion. J Immunol. 2003;170(6):3233–3242. 10.4049/jimmunol.170.6.3233 12626582

[21] Lajqi T , Köstlin‐Gille N , Bauer R , Zarogiannis SG , Lajqi E , Ajeti V , et al. Training vs. tolerance: the Yin/Yang of the innate immune system. Biomedicines. 2023;11(3):766. 10.3390/biomedicines11030766 36979747 PMC10045728

[22] Tozaki T , Kikuchi M , Kakoi H , Hirota KI , Mukai K , Aida H , et al. Profiling of exercise‐induced transcripts in the peripheral blood cells of Thoroughbred horses. J Equine Sci. 2016;27(4):157–164. 10.1294/jes.27.157 27974875 PMC5155134

[23] Mihelić K , Vrbanac Z , Bojanić K , Kostanjšak T , Ljubić BB , Gotić J , et al. Changes in acute phase response biomarkers in racing endurance horses. Animals. 2022;12(21):2993. 10.3390/ani12212993 36359117 PMC9657625

[24] Minamijima Y , Niwa H , Uchida E , Yamamoto K . Comparison of the proteomes in sera between healthy Thoroughbreds and Thoroughbreds with respiratory disease associated with transport using mass spectrometry‐based proteomics. J Equine Sci. 2021;32(1):11–15. 10.1294/jes.32.11 33776535 PMC7984915

[25] Bishop RC , Arrington JV , Wilkins PA , McCoy AM . Alterations in the peritoneal fluid proteome of horses with colic attributed to ischemic and non‐ischemic intestinal disease. Animals. 2025;15(11):1604. 10.3390/ani15111604 40509070 PMC12153689

[26] Cerón JJ , Ortín‐Bustillo A , López‐Martínez MJ , Martínez‐Subiela S , Eckersall PD , Tecles F , et al. S‐100 proteins: basics and applications as biomarkers in animals with special focus on calgranulins (S100A8, A9, and A12). Biology. 2023;12(6):881. 10.3390/biology12060881 37372165 PMC10295460

[27] Powers SK , Duarte J , Kavazis AN , Talbert EE . Reactive oxygen species are signalling molecules for skeletal muscle adaptation. Exp Physiol. 2010;95(1):1–9. 10.1113/expphysiol.2009.050526 19880534 PMC2906150

[28] Margaritelis NV , Paschalis V , Theodorou AA , Kyparos A , Nikolaidis MG . Redox basis of exercise physiology. Redox Biol. 2020;35:101499. 10.1016/j.redox.2020.101499 32192916 PMC7284946

[29] Koju N , Qin ZH , Sheng R . Reduced nicotinamide adenine dinucleotide phosphate in redox balance and diseases: a friend or foe? Acta Pharmacol Sin. 2022;43(8):1889–1904. 10.1038/s41401-021-00838-7 35017669 PMC9343382

[30] Wang J , Ren W , Li Z , Li L , Wang R , Ma S , et al. Plasma lipidomics and proteomics analyses pre‐ and post‐5000 m race in Yili horses. Animals. 2025;15(7):994. 10.3390/ani15070994 40218387 PMC11987874

[31] Tokura Y , Nakayama Y , Fukada S , Nara N , Yamamoto H , Matsuda R , et al. Muscle injury‐induced thymosin β4 acts as a chemoattractant for myoblasts. J Biochem. 2011;149(1):43–48. 10.1093/jb/mvq115 20880960

[32] Bock‐Marquette I , Maar K , Maar S , Lippai B , Faskerti G , Gallyas F Jr , et al. Thymosin beta‐4 denotes new directions towards developing prosperous anti‐aging regenerative therapies. Int Immunopharmacol. 2023;116:109741. 10.1016/j.intimp.2023.109741 36709593

[33] Xing Y , Ye Y , Zuo H , Li Y . Progress on the function and application of thymosin β4. Front Endocrinol. 2021;12:767785. 10.3389/fendo.2021.767785 PMC8724243 34992578

[34] Robinson KA , Sun M , Barnum CE , Weiss SN , Huegel J , Shetye SS , et al. Decorin and biglycan are necessary for maintaining collagen fibril structure, fiber realignment, and mechanical properties of mature tendons. Matrix Biol. 2017;64:81–93. 10.1016/j.matbio.2017.08.004 28882761 PMC5705405

[35] Siqueira RF , Weigel RA , Nunes GR , Mori CS , Fernandes WR . Oxidative profiles of endurance horses racing different distances. Arq Bras Med Vet Zootec. 2014;66(2):455–461. 10.1590/1678-41625760

[36] Wagner EL , Potter GD , Gibbs PG , Eller EM , Scott BD , Vogelsang MM , et al. Copper, zinc‐superoxide dismutase activity in exercising horses fed two forms of trace mineral supplements. J Equine Vet Sci. 2010;30(1):31–37. 10.2527/jas.2010-2871

[37] Allen J , Sun Y , Woods JA . Exercise and the regulation of inflammatory responses. Prog Mol Biol Transl Sci. 2015;135:337–354. 10.1016/bs.pmbts.2015.07.003 26477921

[38] Sugimoto MA , Vago JP , Teixeira MM , Sousa LP . Annexin A1 and the resolution of inflammation: modulation of neutrophil recruitment, apoptosis, and clearance. J Immunol Res. 2016;2016:8239258. 10.1155/2016/8239258 26885535 PMC4738713

[39] Wang S , Song R , Wang Z , Jing Z , Wang S , Ma J . S100A8/A9 in inflammation. Front Immunol. 2018;9:1298. 10.3389/fimmu.2018.01298 29942307 PMC6004386

[40] Zhou Y , Zhang X , Baker JS , Davison GW , Yan X . Redox signaling and skeletal muscle adaptation during aerobic exercise. iScience. 2024;27(5):109643. 10.1016/j.isci.2024.109643 38650987 PMC11033207

[41] Iozzo RV , Schaefer L . Proteoglycan form and function: a comprehensive nomenclature of proteoglycans. Matrix Biol. 2015;42:11–55. 10.1016/j.matbio.2015.02.003 25701227 PMC4859157

[42] Eisner LE , Rosario R , Andarawis‐Puri N , Arruda EM . The role of the non‐collagenous extracellular matrix in tendon and ligament mechanical behavior: a review. J Biomech Eng. 2022;144(5):050801. 10.1115/1.4053086 34802057 PMC8719050

[43] Goh J , Hofmann P , Aw NH , Tan PL , Tschakert G , Mueller A , et al. Concurrent high‐intensity aerobic and resistance exercise modulates systemic release of alarmins (HMGB1, S100A8/A9, HSP70) and inflammatory biomarkers in healthy young men: a pilot study. Transl Med Commun. 2020;5:4. 10.1186/s41231-020-00056-z

[44] Elokda AS , Nielsen DH . Effects of exercise training on the glutathione antioxidant system. Eur J Cardiovasc Prev Rehabil. 2007;14(5):630–637. 10.1097/HJR.0b013e32828622d7 17925621

[45] Parker BL , Kiens B , Wojtaszewski JFP , Richter EA , James DE . Quantification of exercise‐regulated ubiquitin signaling in human skeletal muscle identifies protein modification cross talk via NEDDylation. FASEB J. 2020;34(4):5906–5916. 10.1096/fj.202000075R 32141134

[46] Grzędzicka J , Dąbrowska I , Kiełbik P , Perzyna M , Witkowska‐Piłaszewicz O . Are proteins such as MMp2, IGF1, IL‐13, and IL‐1ra valuable as markers of fitness status in racehorses? A pilot study. Agriculture. 2023;13(11):2134. 10.3390/agriculture13112134

[47] Noda K , Kitagawa K , Miki T , Horiguchi M , Akama TO , Taniguchi T , et al. A matricellular protein fibulin‐4 is essential for the activation of lysyl oxidase. Sci Adv. 2020;6(48):eabc1404. 10.1126/sciadv.abc1404 33239290 PMC7688322

[48] Frystyk J . Exercise and the growth hormone‐insulin‐like growth factor axis. Med Sci Sports Exerc. 2010;42(1):58–66. 10.1249/MSS.0b013e3181b07d2d 20010129

[49] Netea MG , Domínguez‐Andrés J , Barreiro LB , Chavakis T , Divangahi M , Fuchs E , et al. Defining trained immunity and its role in health and disease. Nat Rev Immunol. 2020;20(6):375–388. 10.1038/s41577-020-0285-6 32132681 PMC7186935

[50] Darragh IAJ , O'Driscoll L , Egan B . Exercise training and circulating small extracellular vesicles: appraisal of methodological approaches and current knowledge. Front Physiol. 2021;12:738333. 10.3389/fphys.2021.738333 34777006 PMC8581208

[51] Milczek‐Haduch D , Żmigrodzka M , Witkowska‐Piłaszewicz O . Extracellular vesicles in sport horses: potential biomarkers and modulators of exercise adaptation and therapeutics. Int J Mol Sci. 2025;26(9):4359. 10.3390/ijms26094359 40362597 PMC12073050

[52] Masini A , Tedeschi D , Baragli P , Masini AP , Sighieri C , Lubas G . Exercise‐induced intravascular haemolysis in standardbred horses. Comp Clin Pathol. 2003;12:45–48. 10.1007/s00580-002-0470-y

[53] Pakula PD , Halama A , Al‐Dous EK , Johnson SJ , Filho SA , Suhre K , et al. Characterization of exercise‐induced hemolysis in endurance horses. Front Vet Sci. 2023;10:1115776. 10.3389/fvets.2023.1115776 37180073 PMC10174325

[54] Gegner HM , Naake T , Dugourd A , Müller T , Czernilofsky F , Kliewer G , et al. Pre‐analytical processing of plasma and serum samples for combined proteome and metabolome analysis. Front Mol Biosci. 2022;9:961448. 10.3389/fmolb.2022.961448 36605986 PMC9808085

[55] Blume JE , Manning WC , Troiano G , Hornburg D , Figa M , Hesterberg L , et al. Rapid, deep and precise profiling of the plasma proteome with multi‐nanoparticle protein corona. Nat Commun. 2020;11:3662. 10.1038/s41467-020-17033-7 32699280 PMC7376165

[56] Schoos AM , Nwaru BI , Borres MP . Component‐resolved diagnostics in pet allergy: current perspectives and future directions. J Allergy Clin Immunol. 2021;147(4):1164–1173. 10.1016/j.jaci.2020.12.640 33444632

[57] McDonald RE , Fleming RI , Beeley JG , Bovell DL , Lu JR , Zhao X , et al. Latherin: a surfactant protein of horse sweat and saliva. PLoS One. 2009;4(5):e5726. 10.1371/journal.pone.0005726 19478940 PMC2684629

[58] Espinosa‐López EM , Ortiz‐Guisado B , Diez de Castro E , Durham A , Aguilera‐Tejero E , Gómez‐Baena G . Quantitative proteomics unveils potential plasma biomarkers and provides insights into the pathophysiological mechanisms underlying equine metabolic syndrome. BMC Vet Res. 2025;21(1):425. 10.1186/s12917-025-04879-6 40604814 PMC12217909

