# Supplementary material for: Exercise‐specific plasma proteomic signatures in racehorses: Candidates for training adaptation and peak load monitoring
Source: Equine Vet J. 2025 Dec 29;58(5):1396–412. doi: 10.1002/evj.70146 (PMC13447926; doi:10.1002/evj.70146)
Supplement: Supplementary file 1 [file EVJ-58-1396-s001.pdf]

Equine Veterinary Journal
BEVA

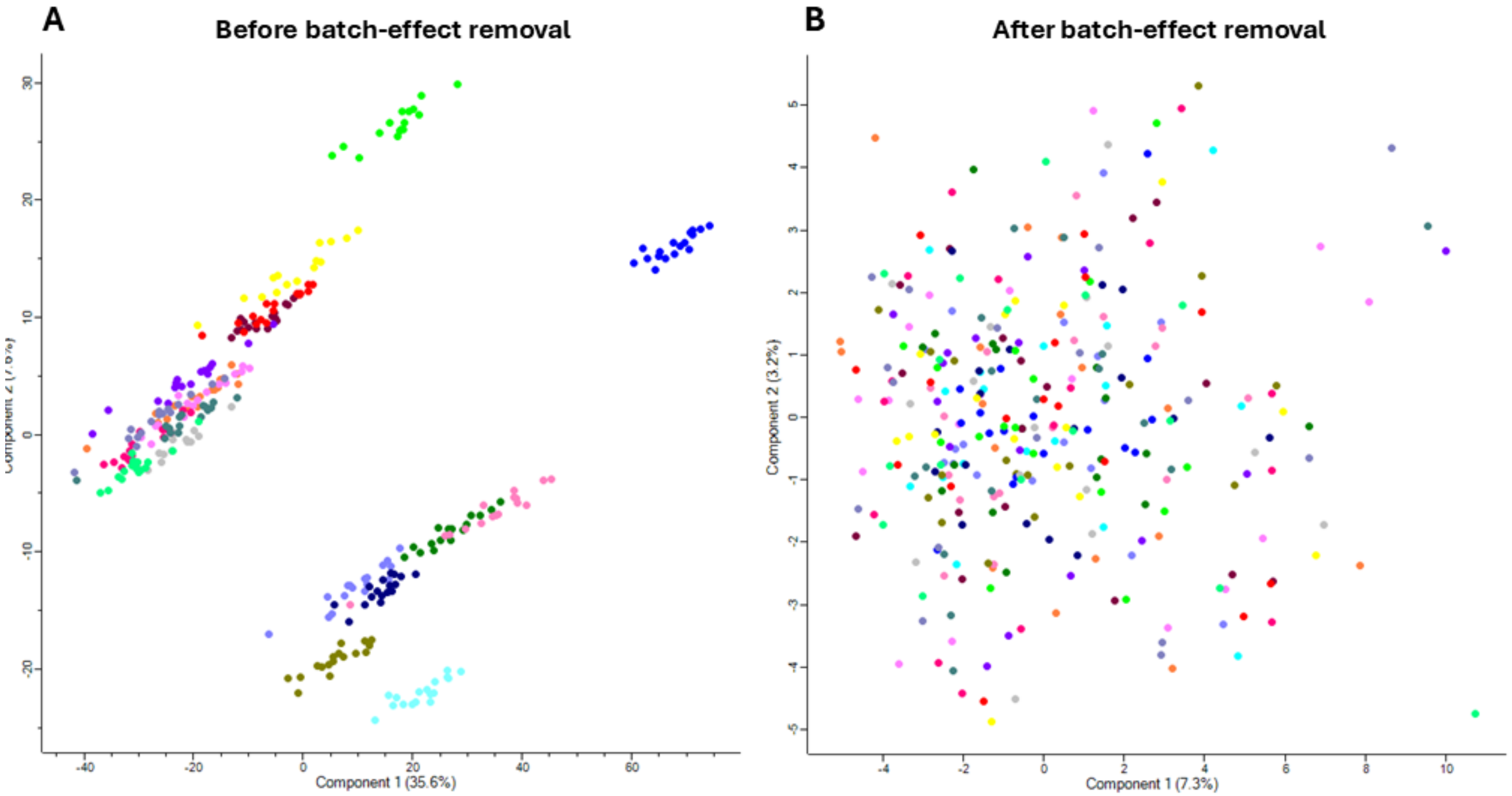

Figure S1. Principal component analysis (PCA) before and after batch-effect removal with ComBat. Points are coloured by TMT set. Panel A shows the uncorrected data, where samples cluster within TMT sets, indicating pronounced batch structure. Panel B shows the data after ComBat normalisation, where TMT-driven separation is largely eliminated and set-specific clustering is no longer apparent. Axes display PC1 and PC2; each point represents one sample.
