# Supplementary material for: Exercise‐specific plasma proteomic signatures in racehorses: Candidates for training adaptation and peak load monitoring
Source: Equine Vet J. 2025 Dec 29;58(5):1396–412. doi: 10.1002/evj.70146 (PMC13447926; doi:10.1002/evj.70146)

Equine Veterinary Journal BEVA

Figure S4. Functional analyses of proteins with altered expression after the initial training session (T1). Panels A–B show upregulated proteins; panels C–D show downregulated proteins. (A) STRING protein–protein interaction (PPI) network for the upregulated set (species background Equus caballus; paired p ≤ 0.05, q ≤ 0.05; median fold change 1.355–2.24). (C) STRING PPI network for the downregulated set (species background Equus caballus; paired p ≤ 0.05, q ≤ 0.05). (B, D) Hierarchical clustering dendrogram of enriched pathways (background Homo sapiens); node size reflects significance (P value).

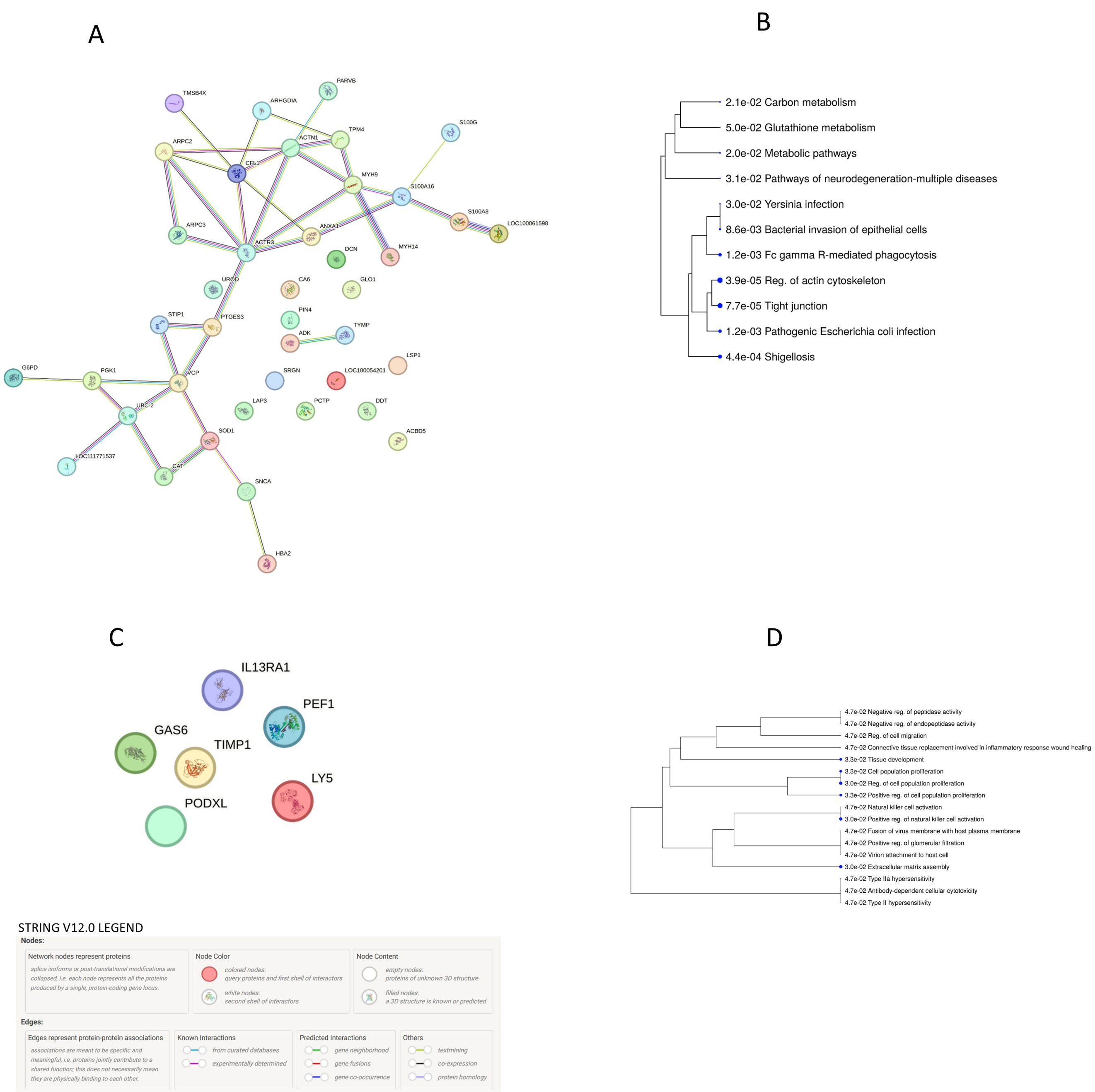

Supplement: Supplementary file 2 [file EVJ-58-1396-s003.pdf]
