# Supplementary material for: Exercise‐specific plasma proteomic signatures in racehorses: Candidates for training adaptation and peak load monitoring
Source: Equine Vet J. 2025 Dec 29;58(5):1396–412. doi: 10.1002/evj.70146 (PMC13447926; doi:10.1002/evj.70146)
Supplement: Supplementary file 3 [file EVJ-58-1396-s004.pdf]

Equine Veterinary Journal BEVA

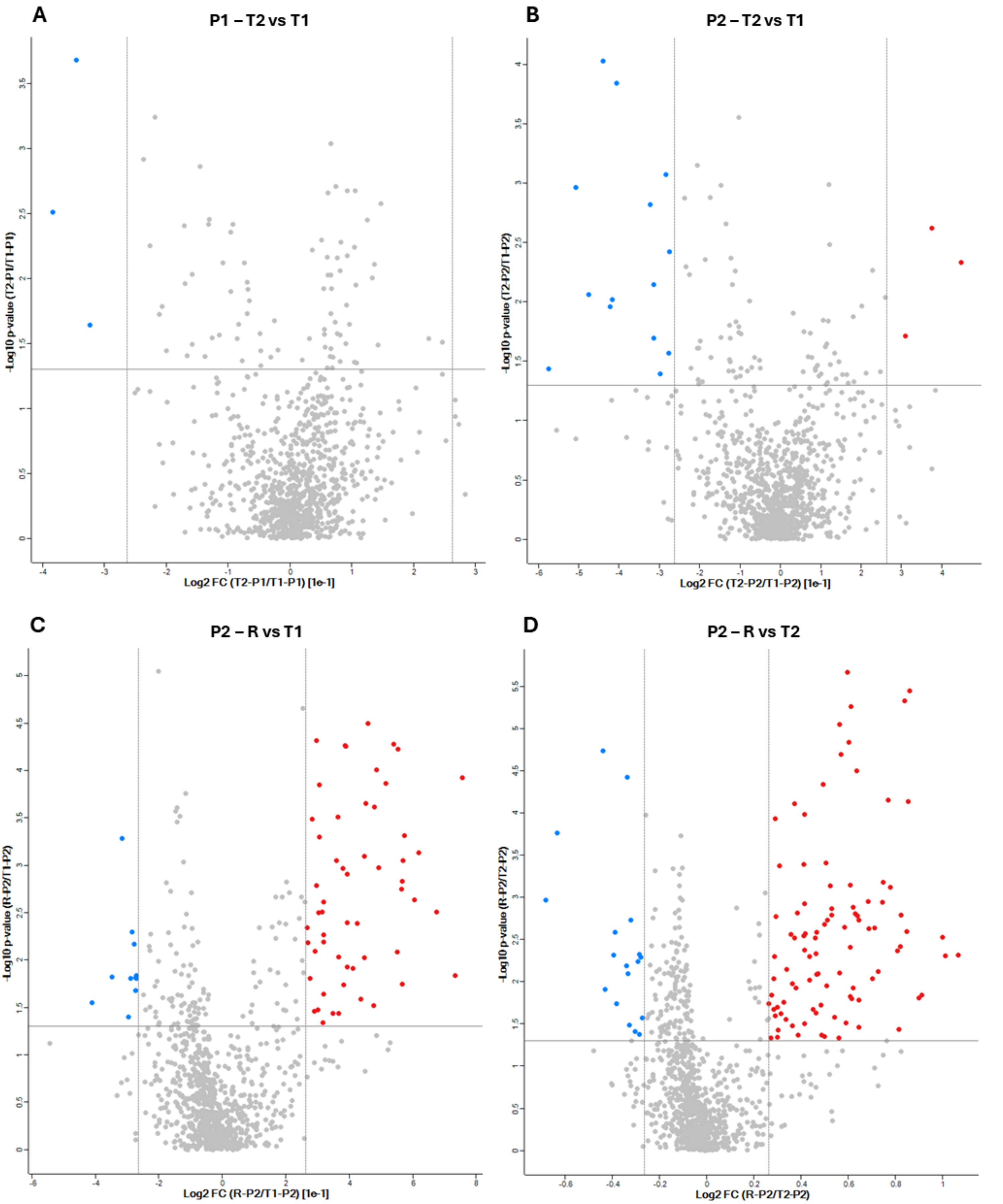

Figure S3. Volcano plots comparing post-exercise protein abundance between sessions. Panel A compares T2 to T1 at p1; Panel B compares T2 to T1 at p2; Panel C compares R to T1 at p2; Panel D compares R to T2 at p2 (T1, initial training; T2, mid-season training; R, race-phase; p1, immediately post-session; p2, 30 min post-session). The x axis shows log2 fold change (FC) and the y axis −log10(FDR q-value). Reference lines indicate statistical significance (horizontal line at 1.30, corresponding to q-value 0.05) and effect size (vertical lines at 0.263, corresponding to 1.2-fold). Proteins meeting both thresholds are highlighted: red for higher abundance in the first-named session of each comparison, blue for lower abundance. Each point represents one protein.
