# Supplementary material for: Exercise‐specific plasma proteomic signatures in racehorses: Candidates for training adaptation and peak load monitoring
Source: Equine Vet J. 2025 Dec 29;58(5):1396–412. doi: 10.1002/evj.70146 (PMC13447926; doi:10.1002/evj.70146)
Supplement: Supplementary file 4 [file EVJ-58-1396-s005.pdf]

Equine Veterinary Journal BEVA

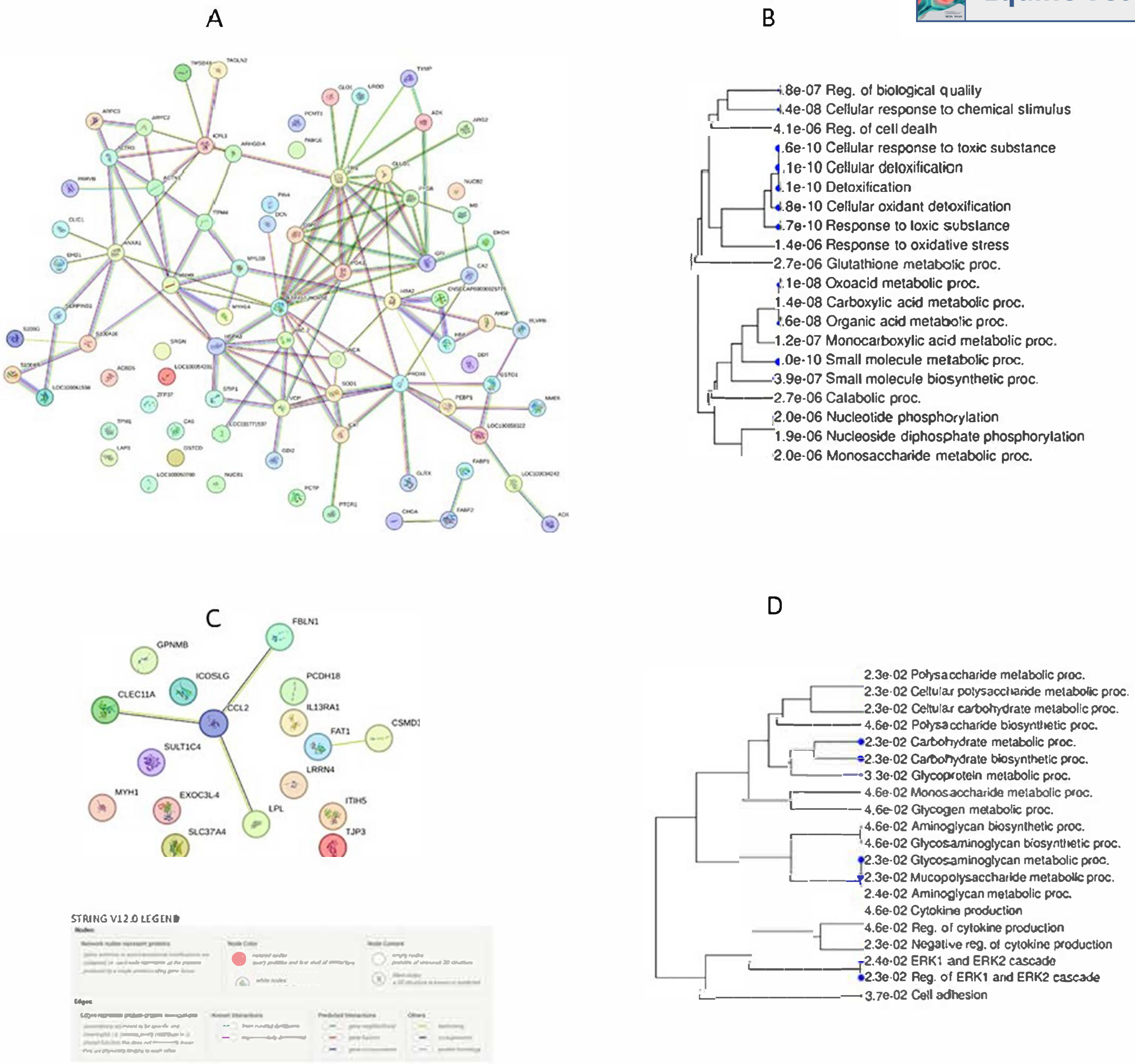

Figure S6. Functional analyses of proteins with altered expression after the race session (R). Panels A–B show upregulated proteins; panels C–D show downregulated proteins. (A) STRING protein–protein interaction (PPI) network for the upregulated set (species background Equus caballus; paired $p \leq 0.05$, $q \leq 0.05$; median fold change 1.355–2.24). (C) STRING PPI network for the downregulated set (species background Homo sapiens; paired $p \leq 0.05$, $q \leq 0.05$). (B, D) Hierarchical clustering dendrogram of enriched pathways (background Homo sapiens); node size reflects pathway significance (P value).
