# Supplementary material for: Exercise‐specific plasma proteomic signatures in racehorses: Candidates for training adaptation and peak load monitoring
Source: Equine Vet J. 2025 Dec 29;58(5):1396–412. doi: 10.1002/evj.70146 (PMC13447926; doi:10.1002/evj.70146)

Equine Veterinary Journal BEVA

Figure S5. Functional analyses of proteins upregulated after the mid-season training session (T2). (A) STRING protein–protein interaction network for the upregulated set (species background Equus caballus; paired $p \leq 0.05$, $q \leq 0.05$; median fold change 1.355–2.24). (B) Hierarchical clustering dendrogram of enriched pathways (background Homo sapiens); node size reflects pathway significance (P value).

A

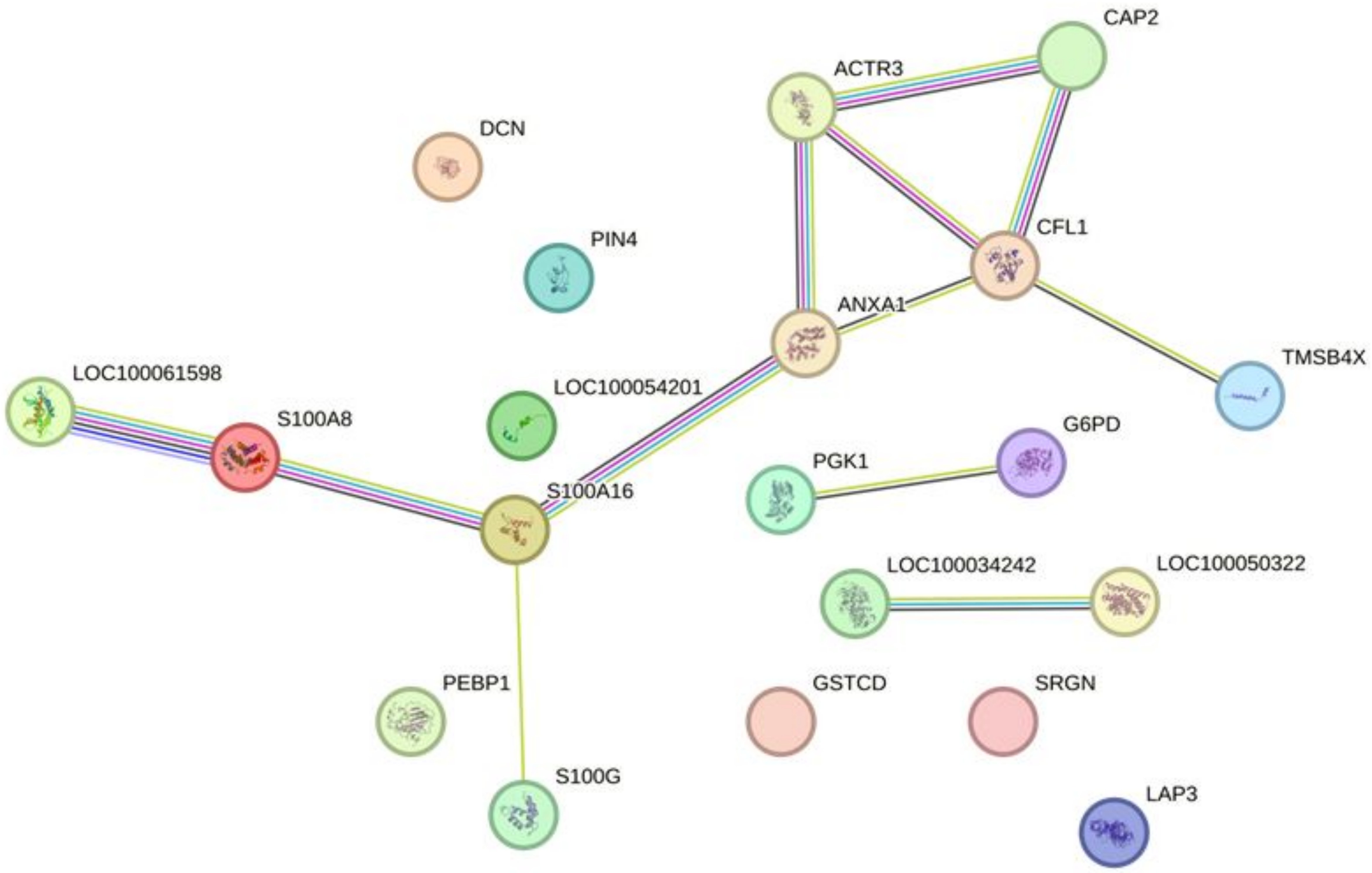

B

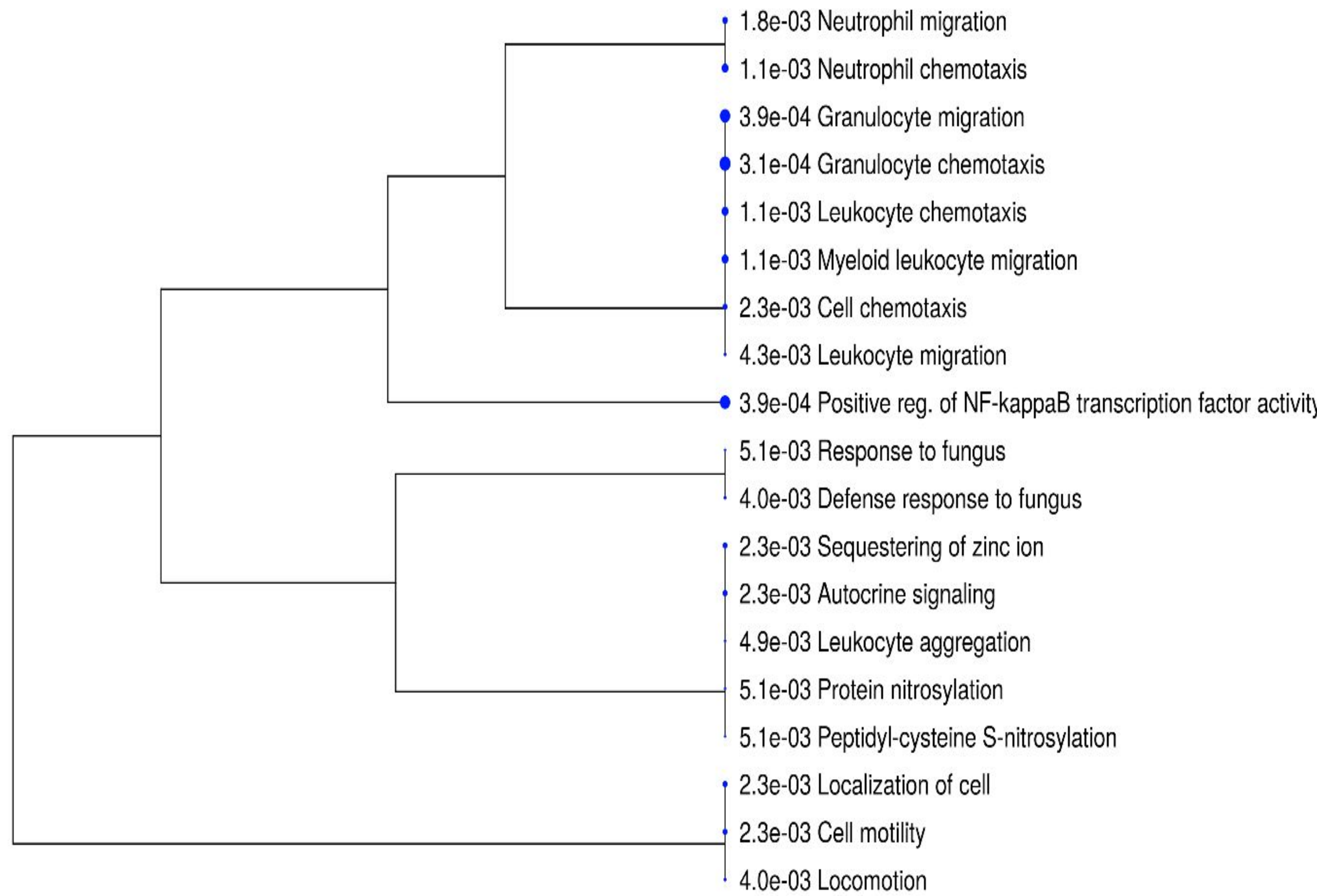

STRING V12.0 LEGEND

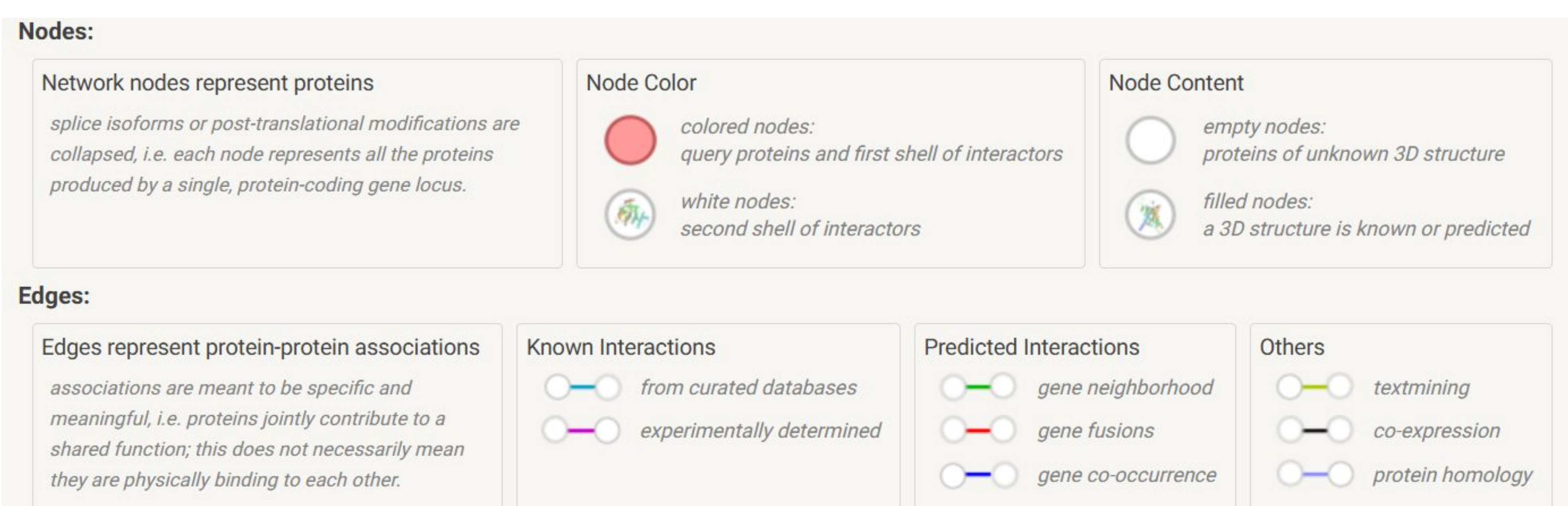

Supplement: Supplementary file 5 [file EVJ-58-1396-s006.pdf]
