# Supplementary material for: Exercise‐specific plasma proteomic signatures in racehorses: Candidates for training adaptation and peak load monitoring
Source: Equine Vet J. 2025 Dec 29;58(5):1396–412. doi: 10.1002/evj.70146 (PMC13447926; doi:10.1002/evj.70146)
Supplement: Supplementary file 6 [file EVJ-58-1396-s009.pdf]

Equine Veterinary Journal
BEVA

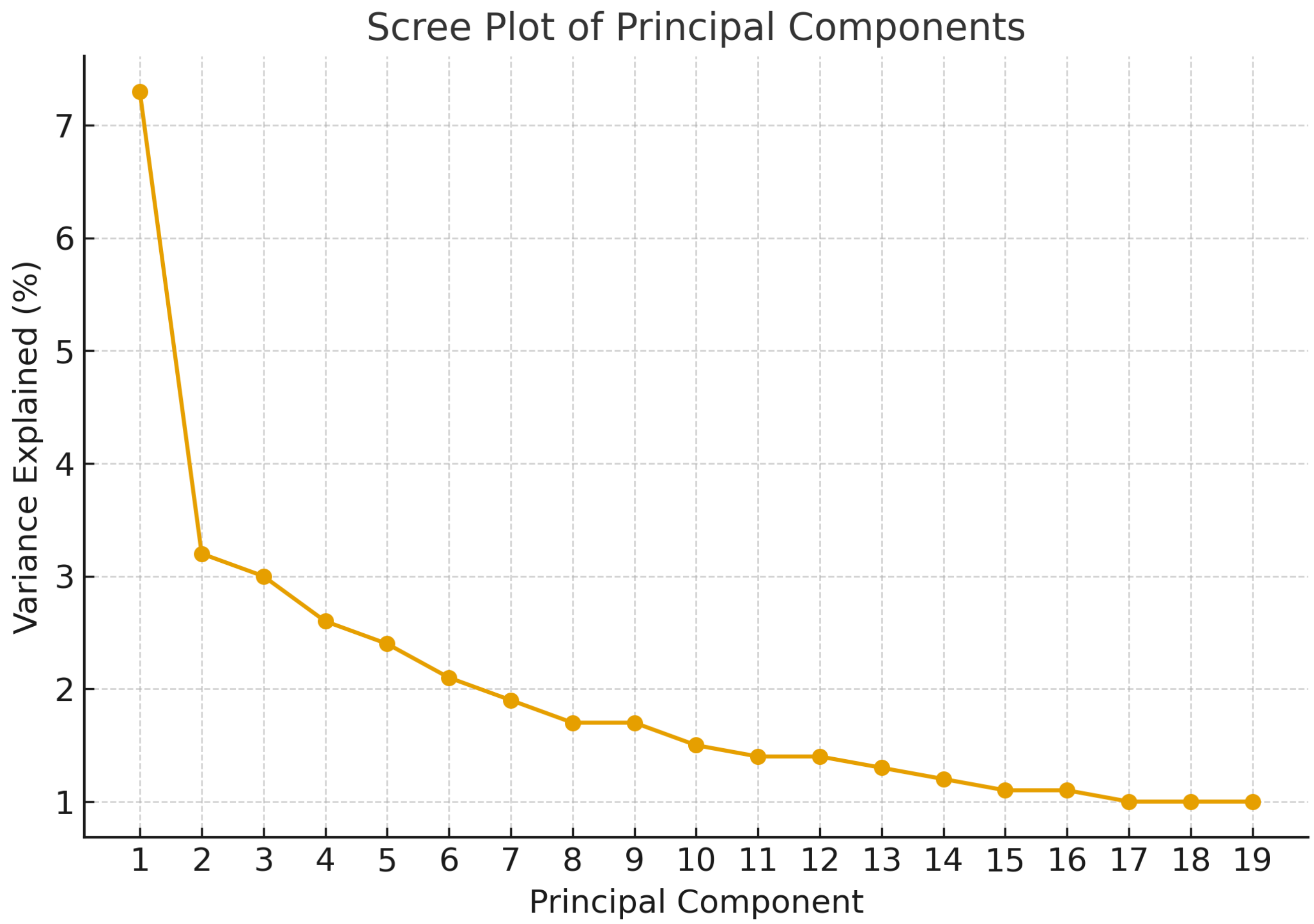

Figure S2. Scree plot of principal components. Percentage of total variance explained by the first 19 PCs calculated from the log_2-transformed, ComBat-normalised plasma-proteomics matrix (all sessions and time points combined).
